# Isolation and Characterization of [D-Leu^1^]microcystin-LY from *Microcystis aeruginosa* CPCC-464

**DOI:** 10.3390/toxins12020077

**Published:** 2020-01-23

**Authors:** Patricia LeBlanc, Nadine Merkley, Krista Thomas, Nancy I. Lewis, Khalida Békri, Susan LeBlanc Renaud, Frances R. Pick, Pearse McCarron, Christopher O. Miles, Michael A. Quilliam

**Affiliations:** 1 Biotoxin Metrology, National Research Council, 1411 Oxford Street, Halifax, NS B3H 3Z1, Canada; Patricia.LeBlanc@nrc-cnrc.gc.ca (P.L.); Nadine.Merkley@nrc-cnrc.gc.ca (N.M.); Krista.Thomas@nrc-cnrc.gc.ca (K.T.); Nancy.Lewis@nrc-cnrc.gc.ca (N.I.L.); khalidabekri@hotmail.com (K.B.); Pearse.McCarron@nrc-cnrc.gc.ca (P.M.); Christopher.Miles@nrc-cnrc.gc.ca (C.O.M.); 2 Department of Biology, University of Ottawa, Ottawa, ON K1N 6N5, Canada; serenaud@gmail.com (S.L.R.); frpick@uOttawa.ca (F.R.P.)

**Keywords:** microcystin, cyanotoxin, structure, PP2A inhibition, liquid chromatography, mass spectrometry, cyanobacteria

## Abstract

[D-Leu^1^]MC-LY (**1**) ([M + H]^+^ *m*/*z* 1044.5673, Δ 2.0 ppm), a new microcystin, was isolated from *Microcystis aeruginosa* strain CPCC-464. The compound was characterized by ^1^H and ^13^C NMR spectroscopy, liquid chromatography–high resolution tandem mass spectrometry (LC–HRMS/MS) and UV spectroscopy. A calibration reference material was produced after quantitation by ^1^H NMR spectroscopy and LC with chemiluminescence nitrogen detection. The potency of **1** in a protein phosphatase 2A inhibition assay was essentially the same as for MC-LR (**2**). Related microcystins, [D-Leu^1^]MC-LR (**3**) ([M + H]^+^ *m*/*z* 1037.6041, Δ 1.0 ppm), [D-Leu^1^]MC-M(O)R (**6**) ([M + H]^+^ *m*/*z* 1071.5565, Δ 2.0 ppm) and [D-Leu^1^]MC-MR (**7**) ([M + H]^+^ *m*/*z* 1055.5617, Δ 2.2 ppm), were also identified in culture extracts, along with traces of [D-Leu^1^]MC-M(O_2_)R (**8**) ([M + H]^+^ *m*/*z* 1087.5510, Δ 1.6 ppm), by a combination of chemical derivatization and LC–HRMS/MS experiments. The relative abundances of **1**, **3**, **6**, **7** and **8** in a freshly extracted culture in the positive ionization mode LC–HRMS were ca. 84, 100, 3.0, 11 and 0.05, respectively. These and other results indicate that [D-Leu^1^]-containing MCs may be more common in cyanobacterial blooms than is generally appreciated but are easily overlooked with standard targeted LC–MS/MS screening methods.

## 1. Introduction

**Figure 1:**
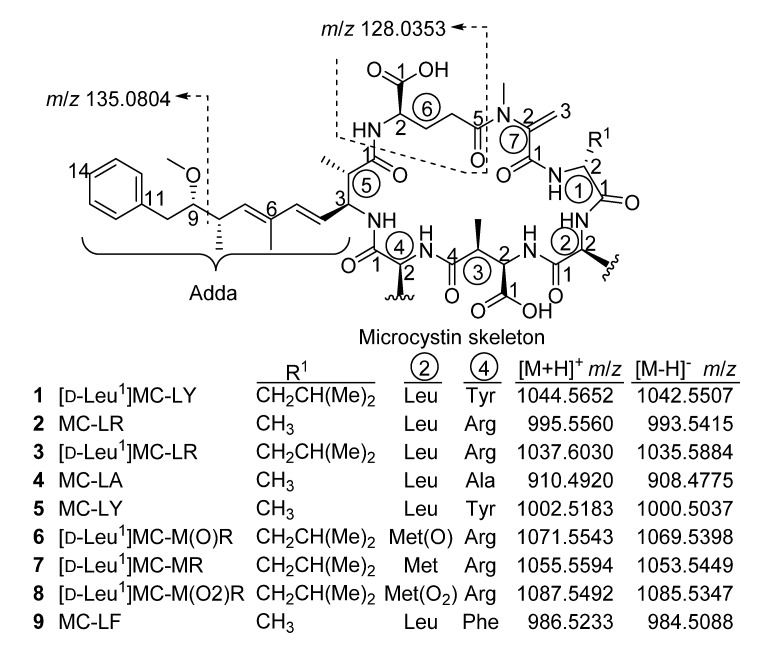
Structures of the microcystins (MCs) mentioned in the text, showing their *m*/*z* values and characteristic mass spectral fragment ions in positive (*m*/*z* 135.0804) and negative (*m*/*z* 128.0353) ionization modes. Numbers in circles indicate the amino acid residue number, while atoms are numbered starting from the carboxyl carbon of each amino acid.

Microcystins (MCs), such as MC-LR (**2**), are cyclic heptapeptide hepatotoxins (Figure 1) produced primarily in cyanobacterial genera such as *Microcystis*, *Dolichospermum* *(Anabaena)*, *Nostoc* and *Planktothrix (Oscillatoria)* [1]. The common feature of MCs is their cyclic structure and possession of several rare, highly conserved amino acids moieties [2]. One of the unusual structural features of MCs is the *β*-amino acid (2*S*,3*S*,8*S*,9*S*)-3-amino-9-methoxy-2,6,8-trimethyl-10-phenyldeca-4(*E*),6(*E*)-dienoic acid (Adda) at position 5 (Figure 1) [3]. Adda^5^ and Glu^6^ appear to be primarily responsible for the characteristic biological activity of MCs [2,3]. Protein phosphatase inhibition is directly related to the toxins’ mechanism of action and animal studies have demonstrated that MCs are potent tumor promoters [1]. To date, the number of identified MCs continues to increase and more than 250 analogues have been characterized [4]. However, due to a lack of standards for these analogues, very few studies have adequately assessed their distribution in natural waters.

As part of feasibility studies for a cyanobacterial matrix reference material [5], a survey of cyanobacterial cultures from Canada was conducted using LC with UV and MS detection. Among the samples analyzed, two *Microcystis aeruginosa* cultures from Saskatchewan and Alberta, CPCC-464 and CPCC-299, showed the presence of a new microcystin tentatively identified as [D-Leu^1^]MC-LY (**1**) [5] together with the previously reported [6,7] and well-characterized [8] [D-Leu^1^]MC-LR (**3**). [D-Leu^1^]MC-LY (**1**) was also tentatively identified recently by LC–HRMS/MS in a cyanobacterial bloom sample from southwestern Ontario, Canada [9], indicating that it may be a significant component of natural cyanobacterial blooms in this and other parts of the world. It is therefore necessary to verify the structure, and to evaluate its toxicity relative to other MCs because limited data are available on the toxicological consequences of varying the amino acid at position 1 and 2.

## 2. Results and Discussion

**Figure 2:**
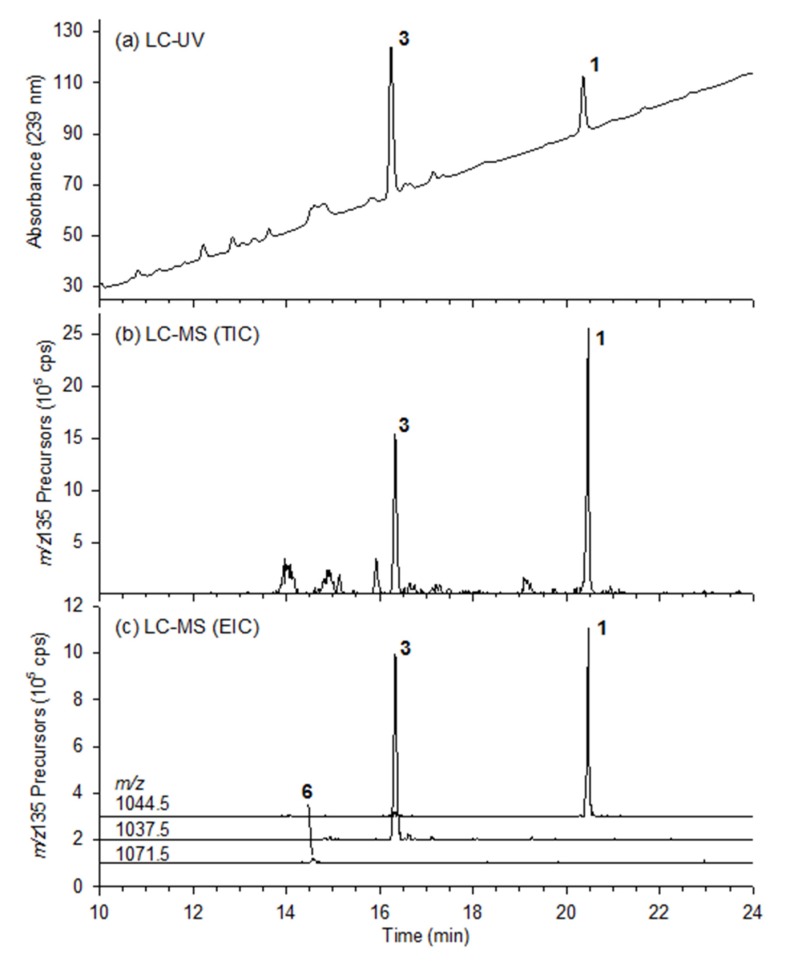
LC–UV–MS analysis of an extract of CPCC-464: (**a**) UV absorbance at 238 nm; (**b**) total ion current chromatogram from *m*/*z* 135 precursor scan; and (**c**) extracted ion chromatograms from *m*/*z* 135 precursor scan.

The analysis of *M. aeruginosa* culture CPCC-464 by LC–UV–MS/MS is shown in Figure 2. A very similar profile was observed with culture CPCC-299, with the only differences being in relative peak areas. The LC–UV chromatogram of CPCC-464 (Figure 2a) showed two major peaks due to **1** and **3**. In the same experiment, the MS was operated with a precursor scan using the *m*/*z* 135 product ion for Adda, which is characteristic of most MCs [10]. Examination of all peaks in the total ion current chromatogram (Figure 2b) revealed three major MCs of interest, **1**, **3** and **6**, with [M+H]^+^ ions at *m*/*z* 1044.5, 1037.5 and 1071.5, respectively. These ions are plotted as extracted ion chromatograms in Figure 2c. LC–HRMS chromatograms with and without mercaptoethanol derivatization are shown in Figures S1 and S2. Compounds **1** and **3** were hypothesized to be [D-Leu^1^]MC-LY and [D-Leu^1^]MC-LR, respectively [5], while the MS characteristics of **6** are consistent with [D-Leu^1^]MC-M(O)R, tentatively identified recently by LC–HRMS/MS in cyanobacterial blooms in Canada and the USA [9,11].

Large scale culturing of CPCC-464 followed by centrifugation provided 188 g of biomass for purification of **1**. This material was extracted with 70% MeOH–H_2_O, then taken through a preparative isolation procedure consisting of a hexane partitioning, C18 LC, LH-20 gel permeation, C18-flash chromatography, and semi-preparative HPLC. The total yield of **1** was 28.7 mg containing a small amount of [D-Leu^1^,D-Glu(OMe)^6^]MC-LY and a trace of what is believed to be [D-Leu^1^,(6*Z*)-Adda^5^]MC-LY (observed by LC–MS selected reaction monitoring modes).

The structure of **1** was elucidated from NMR spectra acquired in CD_3_OH in order to observe the exchangeable amide protons. The proton NMR spectrum had six resonances in the amide region with a profile similar to that of a peptide. Individual spin systems from each amide resonance were identified and assigned using 2D ^1^H-^1^H DIPSI-2 and ^1^H-^1^H COSY correlations (Table 1). Detailed spectra are provided in Figures S3–S10 and an overlay of chemical shifts on the proposed 2-dimensional chemical structure of **1** is shown in Figure S11.

**Figure 3:**
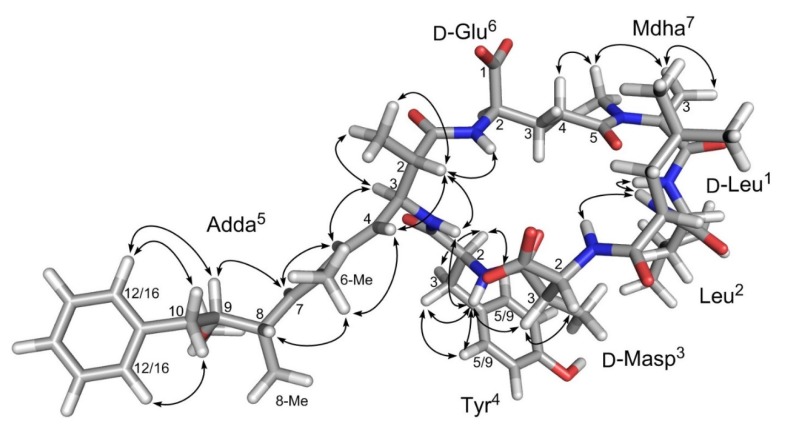
Three-dimensional structure of [D-Leu^1^]MC-LY (**1**), modeled from the solution structure of MC-LR (**2**) [12], showing NoE correlations observed in the ROESY NMR spectrum of **1**.

Carbon assignments were determined indirectly using ^1^H-^13^C HSQC and ^1^H-^13^C HMBC 2D NMR spectra. One carbon resonance was not assigned for Leu^1^ (C1) due to spectral overlap. The Adda unit was assembled with the aid of the HMBC data, which determined the positions of the methyl groups. *Trans*-configuration of the 4,5-double bond is indicated by the large coupling constant between Adda-H4 and -H5 (15.5 Hz) and by the observation of a ROESY correlation between Adda-H4 and Adda-6-Me, and is consistent with the absence of a ROESY correlation between Adda-H4 to Adda-H5. The second double bond was also *trans* as a ROESY correlation was observed between Adda-H5 and Adda-H7 (Figure 3).

The relative stereochemistry of C2 and C3 of Adda was determined from the observation of a ROESY correlation between Adda-H3 and both Adda-H5 and Adda-2-Me, while Adda-H2 showed correlations to Adda-NH, Glu-NH, Adda-2-Me and Adda-H4, indicating that H2 and H3 are on the opposite faces of the Adda plane (Figure 3). This is consistent with the ca. 9.7 Hz coupling constant between Adda-H2 and -H3. The glutamic acid unit (Glu), *N*-methyldehydroalanine (Mdha) and *erthyo*-β-methylaspartic acid (Masp) were identified in a similar manner, and their proton and carbon resonances were very similar to those previously published for **3** [8]. Two leucine units were identified by the similarities of their ^1^H and ^13^C resonances to those in the BMRB database (http://www.bmrb.wisc.edu/ref_info/; accessed September 2011) and those published for **3** [10]. The relative stereochemistry for H2 and H3 of the Masp unit was determined from the absence of Masp-H2 to Masp-3-Me correlation and the presence of a ROESY correlation between Masp-H2 and -H3, which places H2 and H3 on the same side of the plane. The tyrosine unit (Tyr) was assigned from the presence of the two doublets at 6.99 and 6.62 ppm, characteristic of a para-substituted phenyl ring. The aromatic protons, Tyr-H5 and -H9 correlated to a carbon at 36.3 ppm, characteristic of an aromatic amino acid. The accurate mass from LC–HRMS/MS indicated that the substituent on the phenyl ring was a hydroxyl group (Table 2), establishing its identity as Tyr.

**Figure 4:**
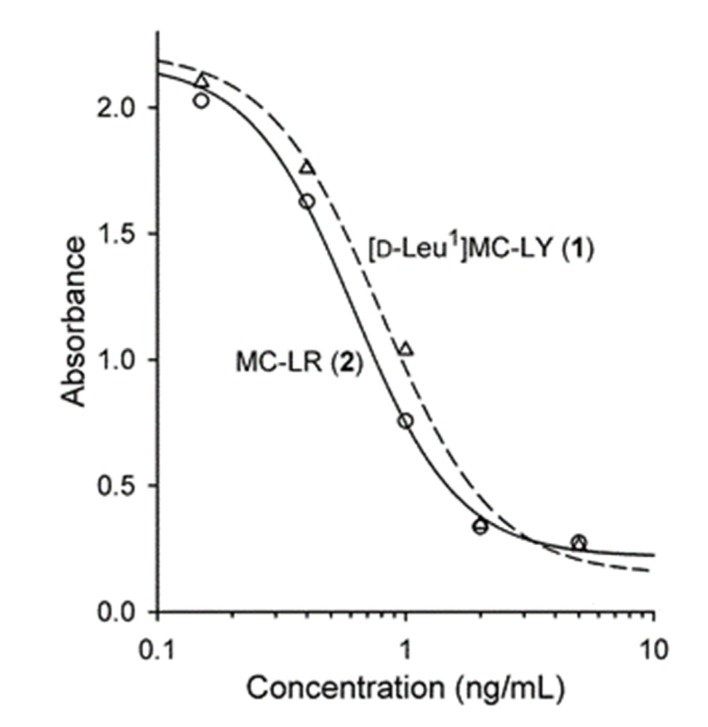
Protein phosphatase 2A (PP2A) inhibition curves for **2** and **1** fitted to a four-parameter logistic curve. The estimated IC_50_ values were 0.62 ng/mL (standard error 0.05) for **2**, and 0.80 ng/mL (SE 0.11) for **1** (0.62 and 0.76 nM, respectively).

The amino acid subunits assigned in the ^1^H-^1^H DIPSI-2, ^1^H-^1^H COSY and ^1^H-^13^C HMBC spectra were linked through correlations observed in the ROESY, NOESY (Figure 3) and HMBC NMR spectra. In the HMBC spectra, a correlation between the *N*-methyl of Mdha and the carbonyl of Glu, and between the Masp-NH and the leucine carbonyl at 174.5 ppm, linked Glu^6^ to Mdha^7^ and Masp^3^ to Leu^2^. Additionally, ROESY correlations were observed between Leu^2^-NH and both Leu^1^-NH and Masp^3^-NH. Furthermore, the Tyr-NH showed ROESY or NOESY correlations to Masp^3^-H3 and Adda^5^-NH, Adda^5^-NH showed correlations to Adda-H4 and Adda-H2, and Adda^5^-H2 showed correlations to Adda-H4, Adda-2-Me and Glu^6^-NH. These correlations show **1** to contain Leu-Leu-Masp-Tyr-Adda-Glu-Mdha, and the molecular formula established from LC–HRMS requires an amide linkage between Mdha^7^ and Leu^1^ moieties. That this linkage is present is demonstrated by the presence of numerous product ions in the HRMS/MS spectrum that are attributable to fragments containing both Leu^1^ and Mdha^7^, such as those at *m*/*z* 169.1334, 197.1283 and 488.2745 (Table 2). The ROESY correlations observed for **1** (Figure 3), especially those between the amide protons, were consistent with those expected based on the established 3-dimensional solution structure for MC-LR [12], which is reported to be very similar to that of [D-Leu^1^]MC-LR (**3**) [8]. Thus, **1** has the same relative stereochemistry as **2** and **3**. This is also supported by the close similarity of the ^13^C NMR chemical shifts of **1** to those reported for [D-Leu^1^]MC-LR (**3**) in the same solvent (Table S1). The fact both **1** and **3** are biosynthesized together by the MC synthetase of *M. aeruginosa* strain CPCC-464, and that **1** was subsequently found to have similar inhibitory potency to MC-LR (**2**) against protein phosphatase 2A (PP2A) (Figure 4), both indicate that **1** has the same absolute stereochemistry as **2** and **3** and that **1** is therefore [D-Leu^1^]MC-LY (Figure 1).

The positive and negative LC–HRMS spectra of **1** were consistent with a molecular formula of C_55_H_77_O_13_N_7_ which, together with the presence of a prominent [M + Na]^+^ adduct ion, a weak neutral loss of *m*/*z* 134.0727, and the late retention time, were consistent with a non-Arg-containing MC. This indicated that **1** contained an extra C_3_H_6_ (42.0470 Da) relative to MC-LY (**5**), and C_9_H_10_O (134.0732 Da) relative to MC-LA (**4**), which differ from **1** only in their amino acids at position 1 (**5**), and at position 1 and 4 (**4**) (Table 2). The negative ion MS/MS spectrum obtained from the FS/DIA (full scan/data independent acquisition) LC–HRMS of **1** showed a prominent product ion at *m/z* 128.0355, consistent with the presence of a MC containing Glu at position 6, and a neutral loss of *m*/*z* 112.0190, consistent with the presence of Masp at position 3 [17]. Careful comparison of the positive ion targeted LC–HRMS/MS spectrum of **1** with those of standards of **4** and **5** (Table 2) showed that product ions in **1** that contained amino acid-1 were consistently heavier by 42.047 Da (Leu vs. Ala) than the corresponding product ions from **5**, and 42.047 (Leu^1^ vs Ala^1^), 92.026 (Tyr^4^ vs Ala^4^) or 134.073 Da (Leu^1^ and Tyr^4^ vs. Ala^1^ and Ala^4^) heavier than the corresponding product ions from **4** that contained amino acid-2, amino acid-4, or both amino acid-1 and -4, respectively (Table 2, Figures S12–S18). Furthermore, the UV spectrum of **1** obtained during LC–UV analysis was identical to that of **5**, and differed from that of **2** (Figure S19), suggesting the presence of Tyr in **1** and **5** in addition to the UV-absorbing chromophores also present in **2** (i.e., Adda^5^ and Mdha^7^). The LC–MS/MS and LC–UV results are therefore entirely consistent with **1** being [D-Leu^1^]MC-LY.A portion of the purified **1** was used to prepare a stock solution. This was quantitated using qNMR [18] and LC with chemiluminescence nitrogen detection (CLND) [19], then accurately diluted with 1:1 MeOH–H_2_O to prepare a reference material (RM) (~7.7 μM). LC–UV analysis of this RM showed the relative concentration of [D-Leu^1^,D-Glu(OMe)^6^]MC-LY to be 3.1%. The putative [D-Leu^1^,(6*Z*)-Adda^5^]MC-LY was below the limit of quantitation in LC–UV, but the relative concentration was estimated be to below 0.5% using HRMS/MS. Because MCs containing (6*Z*)-Adda^5^ or D-Glu(OMe)^6^ do not inhibit protein phosphatases [20], the RM of **1** was used for the PP2A inhibition assay without correcting for impurities. In the PP2A assay, the IC_50_ for a certified RM (CRM) of MC-LR (**2**) was 0.62 nM (0.62 ng/mL), while that for the RM of **1** was 0.76 nM (0.80 ng/mL) (Figure 4). Matthiensen et al. [7] reported that MC-LR (**2**) and [D-Leu^1^]MC-LR (**3**) had similar toxicities to mice when injected intraperitoneally, and that the IC_50_ values of **2** and **3** in a PP1 assay were 3.1 and 4.4 nM, respectively. Similarly, Park et al. [6] independently found that **2** and **3** both had the same IC_50_ value of 0.3 nM in their PP1 assay. Ikehara et al. [21] found that the IC_50_ of MC-LF(**9**), which differs from MC-LY (**5**) only by the absence of a phenolic hydroxyl group on residue-2, was 3-fold higher than that of MC-LR (**2**) (0.096 vs. 0.032 nM) in their PP2A assay. Taken together with the data presented here, these results suggest that the replacement of D-Ala with D-Leu at position 1 in the MC structure has only a minor effect on the toxicity of MCs or on their inhibitory effects on PP1 and PP2A.

Authentic **3** in a cyanobacterial bloom extract from Poplar Island, MD, USA, whose structure has been verified as [D-Leu^1^]MC-LR by purification and NMR analysis [11], had identical retention time and product ion spectra to the peak for **3** in CPCC-464 when analyzed by LC–HRMS/MS, thus verifying its identity as proposed by Hollingdale et al. [5]. LC–HRMS/MS also showed the presence of a minor microcystin with [M + H]^+^ at *m*/*z* 1071.5556 (C_51_H_79_O_13_N_10_S^+^, Δ 1.2 ppm) consistent with [D-Leu^1^]MC-M(O)R (**6**) tentatively identified in bloom samples from south-western Ontario, Canada [9] and Maryland, USA [11], by untargeted LC–HRMS/MS methods and selective chemical oxidation. The identity of this compound in the culture was further verified by targeted LC–HRMS/MS, together with oxidation to its sulfone (**8**) using Oxone [22] (see Figures S20 and S21). In LC–HRMS/MS, **6** showed the neutral loss of methylsulfenic acid (63.9983 Da, CH_3_SOH) characteristic of methyl sulfoxides, and was slowly oxidized to its sulfone by Oxone. The [M + H]^+^ ion for both sulfone-**8**, and sulfoxide-**6** after neutral loss of CH_3_SOH, showed the expected characteristic series of microcystin product ions. Further examination of the LC–HRMS/MS chromatograms revealed the presence of a peak corresponding to [D-Leu^1^]MC-MR (**7**) ([M + H]^+^ *m*/*z* 1055.5608, C_51_H_79_O_12_N_10_S^+^, Δ 1.3 ppm) which showed the expected product ions and which was rapidly converted to **6** by oxidation with Oxone, thus verifying the structures of both **6** and **7**. Trace amounts of the corresponding sulfone (**8**) ([M + H]^+^ *m*/*z* 1087.5491, C_51_H_79_O_14_N_10_S^+^, Δ −0.1 ppm) were also detected in the culture extract, something that was recently also reported by Foss et al. [11] in a cyanobacterial bloom sample, together with **6** and **7**. Microcystins **6–8** from this sample and from *M. aeruginosa* CPCC-464 showed identical retention times and mass spectral characteristics. Methionine sulfoxide analogues of MCs appear to be formed by autoxidation [22], and it appears that the same process can also lead to formation of the corresponding sulfones. The stereochemistry of **6**–**8** cannot be verified by LC–MS methods. However, because **7** is presumably biosynthesized in the culture by the same synthetase that produces **1** and **3**, and that **6** and **8** are autoxidation products of **7**, **6**–**8** can therefore be assumed to have the same stereochemistry as **1** and **3** (Figure 1).

A careful non-targeted LC–MS analysis of a field sample by Foss et al. [11] recently reported more than 20 Leu^1^-containing MCs in a cyanobacterial bloom, with [D-Leu^1^]MC-LR (**3**) as the major component, but no **1** was detected. Including the present study, **1** now appears to have been detected in samples originating from three different locations in Canada [5,9], but so far, nowhere else in the world. Geographical differences in the distribution of microcystins are being reported [23,24]. Leu^1^-containing MCs have been implicated in bird deaths in both Canada and the USA [6,11] and have been reported in samples from cyanobacterial blooms in Brazil and Argentina [7,25,26] as well as in lichens from Argentina, USA, China, Japan, Norway, Sweden, and Finland [25,27]. Leu^1^ variants may be more common and widespread than these studies indicate, as many analyses for MCs are conducted using highly targeted LC–MS/MS methods, and the Leu^1^-containing variants are heavier by 42 Da than the more common (and more commonly targeted) Ala^1^-containing MCs. Both types of variants would be readily detected if they were targeted in the LC–MS/MS method, or if untargeted LC–MS methods were used. Protein phosphatase inhibition assays, or immunoassays with appropriate cross-reactivities [28,29], can also be expected to detect both D-Ala^1^- and D-Leu^1^-containing MCs although they cannot indicate which type of variant is present.

## 3. Conclusions

[D-Leu^1^]MC-LR (**3**) has been reported previously and its structure confirmed by NMR spectroscopy [8]. [D-Leu^1^]MC-LY (**1**) and [D-Leu^1^]MC-M(O)R (**6**) have been tentatively identified by LC–HRMS/MS in a Canadian cyanobacterial bloom sample and in cultures [5,9]. The results presented here firmly establish the identity of **1** and show that it has similar inhibitory potency towards PP2A as MC-LR (**2**). A calibration reference material has been prepared that can be used to identify and quantitate **1** in field samples and cultures. Microcystins containing D-Leu at position 1 may be fairly common in the Americas, and the data presented here and elsewhere suggest these to be only slightly less toxic than their more common D-Ala^1^-containing congeners. It is therefore important to consider the possible presence of a range of D-Leu^1^-containing MCs when analyzing bloom samples.

## 4. Materials and Methods

### 4.1. General Experimental Procedures

Purified **1** (250 µg) was dissolved in 30 µL of CD_3_OH for NMR spectroscopy. NMR spectra were acquired on a Bruker Avance III 600 MHz spectrometer (Bruker Biospin Ltd., Billerica, MA, USA) operating at a ^1^H frequency of 600.28 MHz and ^13^C frequency of 150.94 MHz using TOPSPIN 2.1 acquisition software with a 1.7 mm TXI gradient probe at 277 K. Standard Bruker pulse sequences were used for structure elucidation: one dimensional ^1^H spectrum with composite pulse pre-saturation of water, double quantum filtered ^1^H-^1^H COSY, ^1^H-^1^H DIPSI-2 (mixing time 120 ms), ^1^H-^13^C HSQC, ^1^H-^13^C HMBC (60 and 90 ms delay for long range coupling evolution), ^1^H-^1^H ROESY (mixing time 400 ms) and ^1^H-^1^H NOESY (mixing time 200 ms). A second sample of **1** (1 mg in 210 µL) was prepared in a 3 mm NMR tube for acquisition of the ^13^C spectrum on a Bruker Avance III 700 MHz spectrometer operating at ^1^H frequency of 700.15 MHz and a ^13^C frequency of 176.07 MHz equipped with a 5 mm cryogenically cooled probe at 278 K. The residual ^1^H resonance of CD_3_OH was referenced to 3.31 ppm and the carbon to 48.0 ppm. Data were processed with TOPSPIN 2.1 and analyzed using the NMR assignment software Sparky (T. D. Goddard and D. G. Kneller, SPARKY 3, University of California, San Francisco: https://www.cgl.ucsf.edu/home/sparky/).

The initial survey of cultures for the presence of MCs was performed by LC–UV–MS using an Agilent (Mississauga, ON, Canada) 1200 LC coupled with a SCIEX (Concord, ON, Canada) API 4000 Q-Trap mass spectrometer with UV monitoring at 238 nm and positive electrospray ionization MS, with full scans, *m*/*z* 135 precursor scans, product ion scans, and selected reaction monitoring. The LC column (50 × 2.1 mm; Agilent) was packed with 1.8 µm Zorbax SB-C18 and maintained at 40 °C. The flow rate was 0.3 mL/min, with a gradient of 10%–80% B over 30 min. Solvent A was water and B was 95% acetonitrile, each with 50 mM formic acid and 2 mM ammonium formate.

LC–UV spectra of **1**, **2** and **5** were acquired using an Agilent 1260 LC System with diode array detector (DAD) with UV monitoring at 238 and 210 nm. Separations were on an Acquity HSS T3 1.8 µm column (100 × 2.1 mm; Waters, Milford, MA, USA) held at 40 °C. Isocratic elution was performed with 50% MeOH-H_2_O (0.1% *v/v* trifluoroacetic acid) at 0.25 mL min^−1^ with injection volumes of 5 µL.

LC–HRMS was conducted with a Q Exactive-HF Orbitrap mass spectrometer equipped with a HESI-II heated electrospray ionization interface (ThermoFisher Scientific, Waltham, MA, USA) with an Agilent 1200 G1312B binary pump, G1367C autosampler, and G1316B column oven. Analyses were performed with a 3.5 µm Symmetry Shield C18 column (100 × 2.1 mm; Waters) held at 40 °C with mobile phases A and B of H_2_O and CH_3_CN, respectively, each of which contained formic acid (0.1% *v*/*v*). A linear gradient (0.3 mL min^−1^) was used from 20% to 90% B over 18 min, then to 100% B over 0.1 min, followed by a hold at 100% B (2.9 min), then returned to 20% B over 0.1 min with a hold at 20% B (3.9 min) to equilibrate the column. Injection volume was typically 1–5 µL. In positive ion mode the mass spectrometer was calibrated from *m*/*z* 74–1622, the spray voltage was 3.7 kV, the capillary temperature was 350 °C, and the sheath and auxiliary gas flow rates were 25 and 8 units, respectively, with MS data acquired from 2 to 20 min. Mass spectral data were collected using a combined FS/DIA method. FS data were collected from *m*/*z* 500–1400 using the 60000 resolution setting, an AGC target of 1 × 10^6^ and a max IT of 100 ms. DIA data were collected using the 15000 resolution setting, an AGC target of 2 × 10^5^, max IT set to ‘auto’ and a stepped collision energy of 30, 60 and 80 V. Precursor isolation windows were 62 *m*/*z* wide and centered at *m*/*z* 530, 590, 650, 710, 770, 830, 890, 950, 1010, 1070, 1130, 1190, 1250, 1310, and 1370. DIA chromatograms were extracted for product ions at *m*/*z* 121.1011, 121.0647, 135.0804, 135.1168, 375.1915, 389.2072, 361.1758, 213.0870, 426.2096, 440.2252, 454.2409, 412.1939, 393.2020, 379.1864, 585.3395, 599.3552, and 613.3709. Putative MCs detected using the above FS/DIA method were further probed in a targeted manner using the PRM scan mode with a 0.7 *m*/*z* precursor isolation window, typically using the 30,000 resolution setting, an AGC target of 5 × 10^5^ and a max IT of 400 ms. Typical collision energies were: stepped CE at 30 and 35 eV for MCs with no Arg, and stepped CE at 60, 65 and 70 for MCs with one Arg. LC–MS/MS/MS spectra of *m*/*z* 1071.5 → 1007 for [D-Leu^1^]MC-M(O)R (**6**) were obtained from an LC–HRMS/MS chromatogram run in PRM mode at *m*/*z* 1007.5 with in-source fragmentation energy set at 100 eV. In negative mode, the mass spectrometer was calibrated from *m*/*z* 69–1780 and the spray voltage was −3.7 kV, while the capillary temperature, sheath and auxiliary gas flow rates were the same as for positive mode. Mass spectral data were collected in FS/DIA scan mode as above using a scan range of *m*/*z* 750–1400, a resolution setting of 60000, AGC target of 1 × 10^6^ and max IT of 100 ms. DIA data were collected using a resolution setting of 15,000, AGC target of 2 × 10^5^, max IT set to ‘auto’, and stepped collision energy 65 and 100 V. Isolation windows were 45 *m*/*z* wide and centered at *m*/*z* 772, 815, 858, 902, 945, 988, 1032, 1075, 1118, 1162, 1205, 1248, 1294, 1335, and 1378. DIA chromatograms were extracted for product ions at *m*/*z* 128.0353.

The Oxone oxidations of **6** and **7**, based on Miles et al. [22] were performed by adding 1 μL of Oxone (10 mg/mL) in water to 19 μL of 1:1 MeOH–H_2_O, then 20 μL of culture extract was added with vortex mixing. The reaction was monitored by LC–HRMS/MS after 15 min and then periodically thereafter.

### 4.2. Toxins and Other Materials

Distilled H_2_O was further purified using a UV purification system (ThermoFisher Scientific) or a Milli-Q water purification system (Millipore Ltd., Oakville, ON, Canada). MeOH and CH_3_CN (Optima LC–MS grade) were from ThermoFisher Scientific. Hexanes was from Caledon. Formic acid and trifluoroacetic acid were from Sigma-Aldrich (Oakville, ON, Canada). A certified reference material for **2** (CRM-MCLR (Lot # 20070131)) and in-house reference materials for **4** and **5** were from the National Research Council Canada (Biotoxin Metrology, Halifax, NS, Canada).

### 4.3. Biological Material

*M. aeruginosa* cultures CPCC-464 and CPCC-299 were obtained from the University of Toronto Culture Collection (now the Canadian Phytoplankton Culture Collection housed at the University of Waterloo, ON, Canada). CPCC-464 was isolated from Trampling Lake, Saskatchewan, Canada, July 1998 and deposited by D. Parker as UWOCC#E7. CPCC-299 was isolated from Pretzlaff Pond, Alberta, Canada, August 1990 and deposited by E. Prepas and A. Lam as sample #45-2A. Bulk cultures of CPCC-464 were prepared in two aerated Brite-boxes (250 and 300 L), which are self-contained fiberglass boxes that optimize temperature and light to maximize biomass production. All cultures were grown on BG11 medium [30,31] made using filtered (1 µM) lake water that had pasteurized for 6 h at 85 °C. Light was provided by internally mounted cool white fluorescent tubes shaded with nylon mesh for an approximate intensity of 75–100 µmol m^−2^ s^−1^ on a 14:10 h light:dark cycle. Temperature was maintained at 20 °C and pH was monitored and remained constant at 8.6. When cultures reached late exponential stage, 188 g of wet biomass was harvested using a tangential flow centrifuge (IEC Centra MP-4R CEPA Z41 with an 804S rotor (GMI, Ramsey, MN, USA)) with a flow rate of 2–3 L min^−1^. The biomass was stored at −20 °C. An extract of lyophilized material from a cyanobacterial bloom at Poplar Island, MD, USA, which contained authentic 3 as well as the tentatively identified 6–8, was available from an earlier study [11].

### 4.4. Toxin Isolation from Culture Biomass

Wet cell biomass of CPCC-464 (104.8 g) was extracted four times with 70% MeOH–H_2_O (400 mL). After centrifugation, the supernatants were pooled (1.7 L) and partitioned with hexanes (700 mL). The hexane portion was back-extracted with 85% MeOH–H_2_O (300 mL) and combined with the first extract. The cleaned extract was adjusted to 85% MeOH and partitioned a second time with hexane (300 mL). The combined MeOH–H_2_O extracts were partially evaporated, pre-adsorbed on ~14 g of Waters 55-105µm prep C18 and packed on top of a vacuum liquid chromatography column (4 cm × 11 cm) containing Waters 55–105 µm Prep C18-silica. Fractions were eluted with increasing percentages of MeOH and analyzed by LC–MS. [D-Leu^1^]MC-LY (**1**) was present in fractions containing 40–80% MeOH–H_2_O and further purified on a Sephadex LH-20 column (1.6 cm × 68.0 cm), which was eluted isocratically with MeOH. Fractions containing **1** were combined and subjected to a flash chromatography column (Bakerbond 40 µm C18-silica, 1.5 cm × 22.0 cm) eluted with 55% MeOH–H_2_O. After analyzing the fractions, those containing **1** were purified using a 3 µm Luna C18(2) column (250 × 10 mm; Phenomenex, Torrance, CA, USA) eluted isocratically with 48% CH_3_CN–H_2_O containing 0.05% TFA at 2 mL/min, with UV monitoring at 238 nm. To remove TFA the collected fractions were diluted to 14% CH_3_CN–H_2_O and loaded on Waters Oasis HLB (6 cc, 500 mg) cartridges, washing the acid out with H_2_O and eluting **1** with MeOH.

Purity assessment of the final purified material by LC–MS was carried out on a SCIEX API 4000 mass spectrometer using full scan (*m*/*z* 700–1200) and selected reaction monitoring with positive electrospray ionization. Separations were performed by linear gradient elution on an Agilent 2.7 µm Poroshell 120 SB-C18 column (2.1 × 150 mm) held at 40 °C with mobile phase A: H_2_O, and B: 95% CH_3_CN, each containing 2 mM ammonium formate and 50 mM formic acid. The gradient was 25–75% B over 25 min, increased to 100% B over 2 min, and held for 11 min, at 0.2 mL/min. LC–UV analysis of the pure product was conducted on an Agilent 1290 Infinity LC System with diode array detector (DAD) with UV monitoring at 238 and 210 nm (same LC column and conditions as LC–MS monitoring except mobile phase A: H_2_O, and B: CH_3_CN, each containing 0.1% trifluoroacetic acid).

[D-Leu^1^]MC-LY (**1**): white solid; ^1^H and ^13^C NMR (Table 1); HRMS [M + H]^+^ *m/z* 1044.5660 (calculated for C_55_H_78_O_13_N_7_^+^ 1044.5652 (Δ 0.8 ppm)), LC–HRMS/MS (Table 2); HRMS [M − H]^−^ *m/z* 1042.5515(calculated for C_55_H_76_O_13_N_7_^−^ 1042.5507 (Δ 0.8 ppm)), LC–HRMS/MS (DIA) 1024.5418 (42%), 587.2759 (7), 325.2246 (28), 307.2141 (17), 128.0355 (100). λ_max_ (LC–UV) 233, 280 nm (Figure S19).

### 4.5. Preparation of Reference Material

An aliquot containing [D-Leu^1^]MC-LY (1) (4.3 mg) was evaporated under N_2_ and dissolved in 3.0 mL 90% CD_3_OH–H_2_O. This stock solution was quantitated directly by ^1^H NMR using high purity caffeine as the external calibrant as described previously [18]. A dilution of the stock solution was prepared with 50% MeOH–H_2_O for analysis by LC–UV-CLND [19] using an Agilent 1100 HPLC system with a 1050 UV detector connected to a model 8060 CLND (Antek PAC, Houston, TX, USA). Separations were performed on an Agilent 3.5 µm Poroshell SB-C8 (2.1 × 150 mm) maintained at 40 °C. Isocratic elution was at 0.2 mL/min, using 65% MeOH–H_2_O (0.2% HCOOH) for 1. The external calibrant was also caffeine, with serial dilutions prepared gravimetrically in deionized H_2_O. Caffeine was eluted with 40% MeOH–H_2_O (0.2% HCOOH). The concentration of contaminating [D-Leu^1^,D-Glu(OMe)^6^]MC-LY was measured using the UV detector at 238 nm, with an accurate dilution of the RM of 1 as the calibrant.

After quantitation, the stock solution was quantitatively transferred using 50% high purity degassed MeOH–H_2_O to a calibrated volumetric flask, then diluted to the mark with the same. The solution was packaged under argon in flame sealed ampoules using an automatic ampouling machine (Cozzolli, Model FPS1-SS-428, NJ, USA), then stored at −80 °C.

### 4.6. Protein Phosphatase Inhibition Assay

Ampoules of the RM of **1**, along with the CRM of **2** (CRM-MCLR), were sent to Abraxis LLC (Warminster, PA, USA) for evaluation of toxicity. PP2A assays were performed using the microcystin-PP2A plate kit according to the kit’s standard procedures [32].

## Figures and Tables

**Table 1:** NMR spectroscopic data for [D-Leu^1^]MC-LY (**1**) in CD_3_OH*^a^*.

Unit	Position	δc, Type	δ_H_, Multiplicity (*J* in Hz)	HMBC
D-Leu^1^	1	ND		
	2	52.0, CH	4.44, m	
	2-NH		7.89, d (7.7)	
	3a	39.2, CH_2_	1.62, m	4
	3b		1.33, m	
	4	24.8, CH	1.57, m	
	4-Me	19.9, CH_3_	0.83, d (6.3)	
	5	22.6, CH_3_	0.83, d (6.3)	
Leu^2^	1	174.5, C		
	2	53.8, CH	4.23, ddd (10.4, 6.6, 4.0)	
	2-NH		8.28, d (6.5)	1
	3a	39.8, CH_2_	1.92, ddd (13.7, 12.0, 4.0)	2,5/6
	3b		1.51, ddd (13.7, 10.4, 3.8)	
	4	24.5, CH	1.76, m	
	4-Me	20.0, CH_3_	0.88, d (6.6)	
	5	22.8, CH_3_	0.88, d (6.6)	
D-Masp^3^	1	175.6, C		
	2	54.6, CH	4.60, dd (9.0, 3.8)	1,4
	2-NH		7.72, d (9.0)	Leu^2^-1
	3	40.7, CH	3.04, dq (7.2, 3.8)	
	3-Me	13.8, CH_3_	0.86, d (7.2)	2,4
	4	177.4, C		
Tyr^4^	1	170.6, C		
	2	54.1, CH	4.35, ddd (11.7, 9.3, 3.3)	1
	2-NH		8.89, d (9.3)	
	3a	36.3, CH_2_	3.29, m	1
	3b		2.54, dd (14.1, 11.7)	2,4,5/9
	4	128.3, C		
	5/9	130.2, CH	6.99, d (8.5)	3,7,5/9
	6/8	115.0, CH	6.62, d (8.5)	4,7,6/8
	7	156.4, C		
Adda^5^	1	176.0, C		
	2	44.1, CH	2.76, m	
	2-Me	14.8, CH_3_	1.08, d (6.9)	1,2,3
	3	55.5, CH	4.70, ~q (9.7)	1,2,4,5
	3-NH		7.36, d (9.2)	
	4	125.2, CH	5.46, dd (15.5, 9.0)	6
	5	138.0, CH	6.32, d (15.5)	3,7,6-Me
	6	132.8, C		
	6-Me	11.8, CH_3_	1.63, s	5,6,7
	7	136.2, CH	5.49, d (10.1)	5,6,8,9,6-Me
	8	36.5, CH	2.61, m	
	8-Me	15.5, CH_3_	1.03, d (6.7)	7,8,9
	9	87.2, CH	3.27, m	
	9-OMe	57.6, CH_3_	3.24, s	9
	10a	37.7, CH_2_	2.83, dd (13.9, 4.7)	12/16
	10b		2.68, dd (13.9, 7.4)	9,11,12/16
	11	139.4, C		
	12/16	129.5, CH	7.19, m	10,14,12/16
	13/15	128.1, CH	7.25, t (7.6)	11,13/15
	14	125.9, CH	7.17, m	
D-Glu^6^	1	174.8, C		
	2	53.3, CH	4.31, ~q (7.2)	1,3
	2-NH		7.55, brs	
	3a	27.0, CH_2_	2.10, m	
	3b		1.79, m	
	4a	32.0, CH_2_	2.72, m	
	4b		2.59, m	
	5	175.2, C		
Mdha^7^	1	165.5, C		
	2	145.1, C		
	2-NMe	37.3, CH_3_	3.36, s	2, D-Glu-5
	3*E*	113.3, CH_2_	5.84, s	1
	3*Z*		5.43, s	1,2

*^a^*s, singlet; d, doublet; t, triplet; q, quartet; m, multiplet; br, broad; ND, not detected.

**Table 2:** Reported product ions for MC-LA (**4**), with their exact *m*/*z* values and amino acid origins, and the corresponding product ions and their mass differences relative to the reported values for MC-LA, observed during LC–HRMS/MS analysis of MC-LA (**4**), MC-LY (**5**), and [D-Leu^1^]MC-LY (**1**) in the positive ionization mode*^a^*.

AA Origin							Reported for MC-LA (4)		MC-LA (4)		MC-LY (5)		[D-Leu^1^]MC-LY (1)	
1	2	3	4	5	6	7	Calc. *m*/*z*	Formula	*m*/*z*	Diff.	*m*/*z*	Diff.	*m*/*z*	Diff.
							44.0495	C_2_H_6_N^+^	ND	N/A	136.0757	92.0262	136.0756	92.0261
							56.0495	C_3_H_6_N^+^	56.0497	0.0002	56.0497	0.0002	56.0497	0.0002
							84.0444	C_4_H_6_NO^+^	84.0443	-0.0001	84.0444	0.0000	84.0444	0.0000
							86.0964	C_5_H_12_N^+^	86.0964	0.0000	86.0964	0.0000	86.0964	0.0000
							103.0542	C_8_H_7_^+^	103.0541	-0.0001	103.0542	0.0000	103.0542	0.0000
							107.0855	C_8_H_11_^+^	107.0854	-0.0001	107.0855	0.0000	107.0855	0.0000
							127.0866	C_6_H_11_N_2_O^+^	127.0865	-0.0001	127.0866	0.0000	169.1334	42.0468
							135.0804	C_9_H_11_O^+^	135.0803	-0.0001	135.0804	0.0000	135.0803	-0.0001
							135.1168	C_10_H_15_^+^	135.1166	-0.0002	135.1167	-0.0001	135.1166	-0.0002
							155.0815	C_7_H_11_N_2_O_2_^+^	155.0813	-0.0002	155.0813	-0.0002	197.1283	42.0468
							163.1117	C_11_H_15_O^+^	163.1115	-0.0002	163.1117	0.0000	163.1115	-0.0002
							173.0921	C_7_H_13_N_2_O_3_^+^	173.0917	-0.0004	265.1180	92.0259	265.1179	92.0258
							195.0764	C_9_H_11_N_2_O_3_^+^	195.0763	-0.0001	195.0764	0.0000	195.0763	-0.0001
							213.0870	C_9_H_13_N_2_O_4_^+^	213.0868	-0.0002	213.0869	-0.0001	213.0868	-0.0002
							218.1135	C_8_H_16_N_3_O_4_^+^	218.1133	-0.0002	310.1393	92.0258	310.1392	92.0257
							265.1587	C_19_H_21_O^+^	265.1581	-0.0006	265.1583	-0.0004	265.1581	-0.0006
							268.1656	C_13_H_22_N_3_O_3_^+^	268.1650	-0.0006	268.1652	-0.0004	310.2119	42.0463
							292.1543	C_16_H_22_NO_4_^+^	292.1538	-0.0005	292.1541	-0.0002	292.1540	-0.0003
							314.1710	C_14_H_24_N_3_O_5_^+^	314.1705	-0.0005	406.1967	92.0257	406.1965	92.0255
							331.1976	C_14_H_27_N_4_O_5_^+^	331.1970	-0.0006	423.2230	92.0254	423.2229	92.0253
							375.1914	C_20_H_27_N_2_O_5_^+^	375.1906	-0.0008	375.1912	-0.0002	375.1907	-0.0007
							385.2082	C_17_H_29_N_4_O_6_^+^	385.2073	-0.0009	477.2359	92.0277	519.2807	134.0725
							402.2347	C_17_H_32_N_5_O_6_^+^	402.2342	-0.0005	494.2602	92.0255	536.3072	134.0725
							446.2286	C_23_H_32_N_3_O_6_^+^	446.2276	-0.0010	446.2283	-0.0003	488.2745	42.0459
							468.2453	C_21_H_34_N_5_O_7_^+^	468.2443	-0.0010	560.2707	92.0254	602.3180	134.0727
							485.2718	C_21_H_37_N_6_O_7_^+^	485.2708	-0.0010	577.2976	92.0258	619.3447	134.0729
							509.2646	C_29_H_37_N_2_O_6_^+^	509.2637	-0.0009	509.2640	-0.0006	509.2640	-0.0006
							559.3126	C_29_H_43_N_4_O_7_^+^	559.3117	-0.0009	559.3157	0.0031	601.3610	42.0484
							580.3017	C_32_H_42_N_3_O_7_^+^	580.3008	-0.0009	580.3010	-0.0007	622.3479	42.0462
							597.2879	C_26_H_41_N_6_O_10_^+^	597.2872	-0.0007	689.3134	92.0255	731.3605	134.0726
							693.3858	C_38_H_53_O_8_N_4_^+^	693.3854	-0.0004	693.3854	-0.0004	735.4322	42.0464
							758.4083	C_37_H_56_N_7_O_10_^+^	758.4076	-0.0007	850.4350	92.0267	892.4804	134.0721
							759.3923	C_37_H_55_N_6_O_11_^+^	759.3917	-0.0006	851.4190	92.0267	893.4652	134.0729
							776.4189	C_37_H_58_N_7_O_11_^+^	776.4187	-0.0002	868.4445	92.0256	910.4906	134.0717
							910.4920	C_46_H_68_N_7_O_12_^+^	910.4908	-0.0012	1002.5175	92.0255	1044.5634	134.0714

*^a^* Reported fragments, their amino acid origins and calculated *m*/*z* for MC-LA based on data from Bortoli and Volmer 2014; Mayumi et al. 2006; Miles et al. 2013; Stewart et al. 2018 [13,14,15,16]; black squares indicate amino acids contributing to each product ion; gray squares indicate neutral loss of 134.0732 Da from Adda^5^; Diff., difference from calculated *m*/*z* for the corresponding product ion in MC-LA; ND, not detected.

## Supplementary Materials

The following are available online at https://www.mdpi.com/2072-6651/12/2/77/s1. Figure S1: LC–HRMS of CPCC-464 with thiol derivatization (positive mode), Figure S2: LC–HRMS of CPCC-464 (positive and negative modes), Figure S3: ^1^H NMR Spectrum of [Leu^1^]MC-LY (**1**) in CD_3_OH, Figure S4: COSY NMR Spectrum of [Leu^1^]MC-LY (**1**) in CD_3_OH, Figure S5: DIPSI NMR Spectrum of [Leu^1^]MC-LY (**1**) in CD3OH, Figure S6: HSQC NMR Spectrum of [Leu^1^]MC-LY (**1**) in CD_3_OH, Figure S7: HMBC NMR Spectrum of [Leu^1^]MC-LY (**1**) in CD_3_OH, Figure S8: ^13^C NMR Spectrum of [Leu1]MC-LY (**1**) in CD_3_OH, Figure S9: ROESY NMR Spectrum of [Leu1]MC-LY (**1**) in CD_3_OH, Figure S10: NOESY NMR Spectrum of [Leu^1^]MC-LY (**1**) in CD_3_OH, Figure S11: ^1^H and ^13^C chemical shifts overlaid on the structure of [Leu^1^]MC-LY (**1**), Figure S12: MS/MS spectra of MC-LA (**4**), MC-LY (**5**) and [Leu^1^]MC-LY (**1**), Figures S13–S18: MS/MS spectra of MC-LA (**4**), MC-LY (**5**) and [Leu^1^]MC-LY (**1**) (expanded), Figure S19: UV spectra of MC-LR (**2**), MC-LY (**5**) and [Leu^1^]MC-LY (**1**), Figure S20: LC–HRMS of CPCC-464 with Oxone oxidation (positive mode), Figure S21: MS/MS spectra of [Leu1]MC-M(O)R (**6**) and [Leu^1^]MC-M(O)R (**7**), Table S1: Comparison of the ^13^C chemical shift assignments for 1 in CD_3_OH with those reported for 3 in CD_3_OD.
