# Supplementary material for: Isolation and Characterization of [D-Leu1]microcystin-LY from Microcystis aeruginosa CPCC-464
Source: Toxins (Basel). 2020 Jan 23;12(2):77. doi: 10.3390/toxins12020077 (PMC7076857; doi:10.3390/toxins12020077)
Supplement: Supplementary file 1 [file toxins-12-00077-s001.pdf]

Supplementary Material: Isolation and characterization of [D-Leu[1]]microcystin-LY from *Microcystis aeruginosa* CPCC-464

Patricia LeBlanc, Nadine Merkley, Krista Thomas, Nancy I. Lewis, Khalida Békri, Susan LeBlanc Renaud, Frances R. Pick, Pearse McCarron, Christopher O. Miles and Michael A. Quilliam

Supplementary Materials

Figure S1	LC–HRMS of CPCC-464 with thiol derivatization (positive mode)	S2
Figure S2	LC–HRMS of CPCC-464 (positive & negative modes)	S3
Figure S3	^{1}H NMR Spectrum of [Leu[1]]MC-LY (**1**) in CD_3OH	S4
Figure S4	COSY NMR Spectrum of [Leu[1]]MC-LY (**1**) in CD_3OH	S5
Figure S5	DIPSI NMR Spectrum of [Leu[1]]MC-LY (**1**) in CD_3OH	S6
Figure S6	HSQC NMR Spectrum of [Leu[1]]MC-LY (**1**) in CD_3OH	S7
Figure S7	HMBC NMR Spectrum of [Leu[1]]MC-LY (**1**) in CD_3OH	S8
Figure S8	^{13}C NMR Spectrum of [Leu[1]]MC-LY (**1**) in CD_3OH	S9
Figure S9	ROESY NMR Spectrum of [Leu[1]]MC-LY (**1**) in CD_3OH	S10
Figure S10	NOESY NMR Spectrum of [Leu[1]]MC-LY (**1**) in CD_3OH	S11
Figure S11	^{1}H and ^{13}C chemical shifts overlaid on the structure of [Leu[1]]MC-LY (**1**)	S12
Figure S12	MS/MS spectra of MC-LA (**4**), MC-LY (**5**) and [Leu[1]]MC-LY (**1**)	S13
Figure S13	MS/MS spectra of MC-LA (**4**), MC-LY (**5**) and [Leu[1]]MC-LY (**1**) (expanded)	S14
Figure S14	MS/MS spectra of MC-LA (**4**), MC-LY (**5**) and [Leu[1]]MC-LY (**1**) (expanded)	S15
Figure S15	MS/MS spectra of MC-LA (**4**), MC-LY (**5**) and [Leu[1]]MC-LY (**1**) (expanded)	S16
Figure S16	MS/MS spectra of MC-LA (**4**), MC-LY (**5**) and [Leu[1]]MC-LY (**1**) (expanded)	S17
Figure S17	MS/MS spectra of MC-LA (**4**), MC-LY (**5**) and [Leu[1]]MC-LY (**1**) (expanded)	S18
Figure S18	MS/MS spectra of MC-LA (**4**), MC-LY (**5**) and [Leu[1]]MC-LY (**1**) (expanded)	S19
Figure S19	UV spectra of MC-LR (**2**), MC-LY (**5**) and [Leu[1]]MC-LY (**1**)	S20
Figure S20	LC–HRMS of CPCC-464 with Oxone oxidation (positive mode)	S21
Figure S21	MS/MS spectra of [Leu[1]]MC-M(O)R (**6**) and [Leu[1]]MC-M(O)R (**7**)	S22
Table S1	Comparison of ^{13}C chemical shifts for **1** (CD_3OH) and **3** in (CD_3OD)	S23

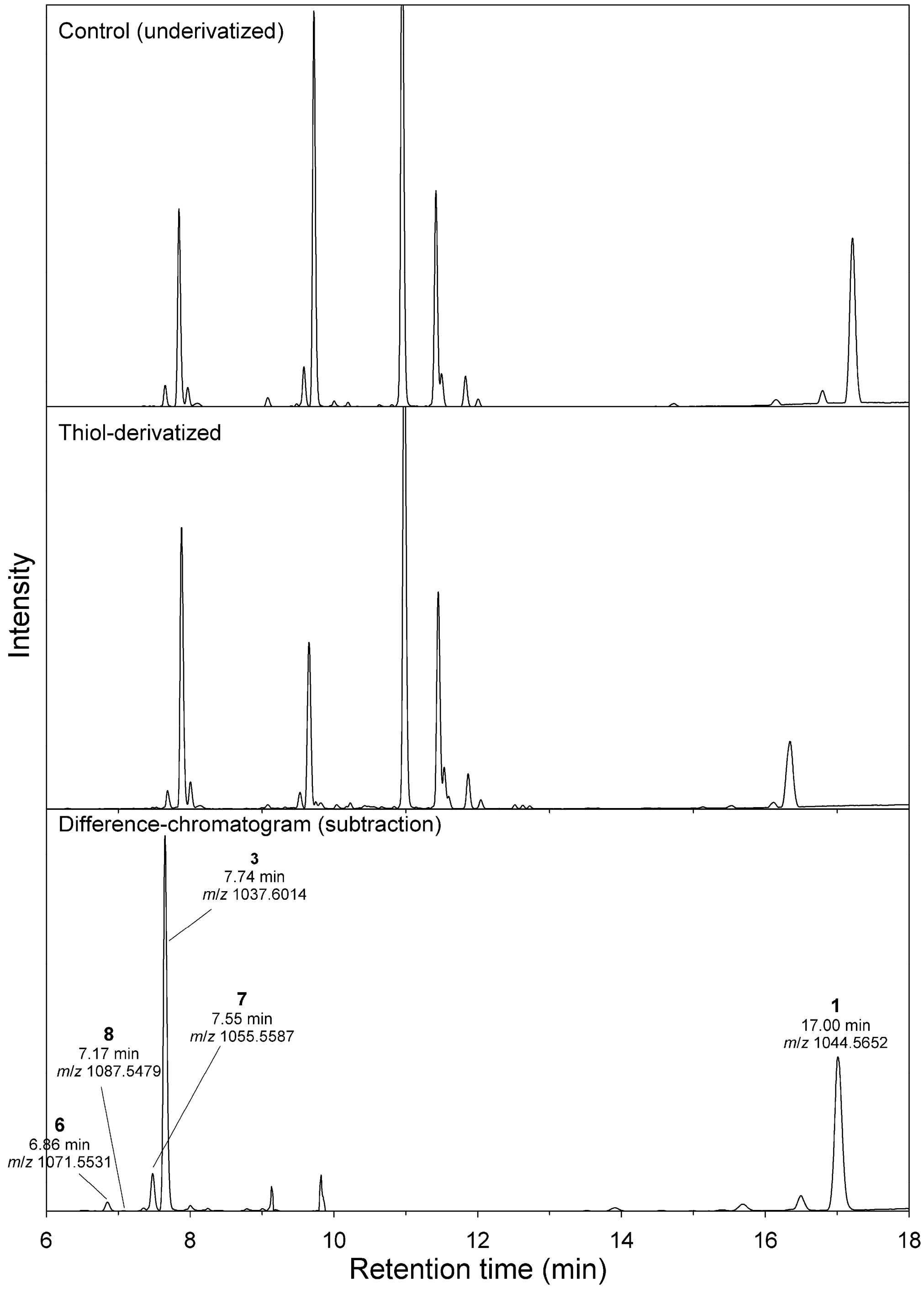

Figure S1. Positive ionization LC-HRMS chromatograms of an extract of *M. aeruginosa* CPCC-464 before (top) and after (middle) derivatization with mercaptoethanol. The bottom chromatogram shows a subtraction of the derivatized from the underivatized chromatograms, and shows the identities (Figure 1), retention times, and *m*/*z* for $[M + H]^+$ of microcystins referred to in the text.

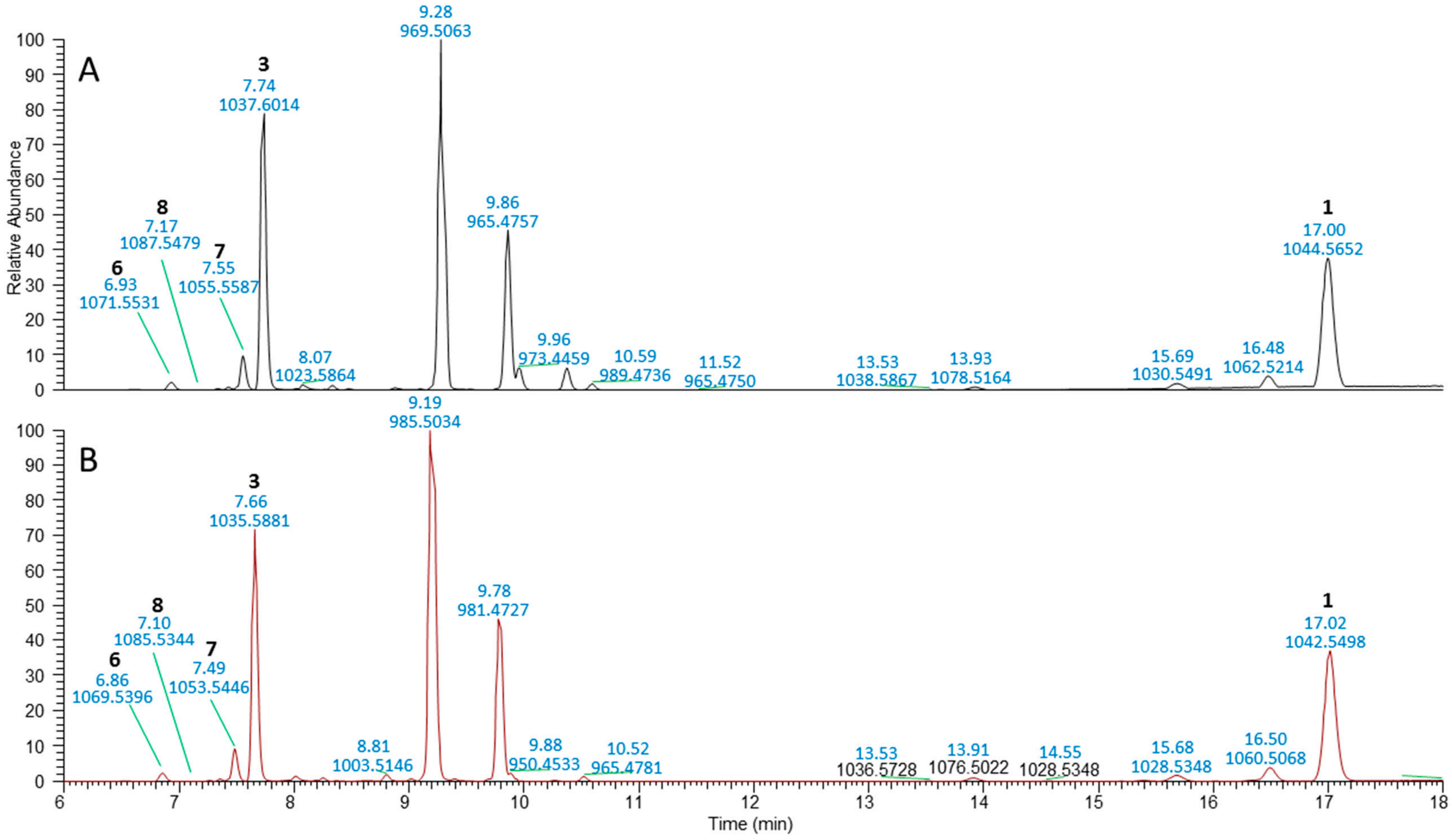

Figure S2. LC-HRMS full scan chromatograms obtained in positive (**A**) and negative (**B**) ionization modes (*m*/*z* 900–1100) of an extract of *M. aeruginosa* CPCC-464.

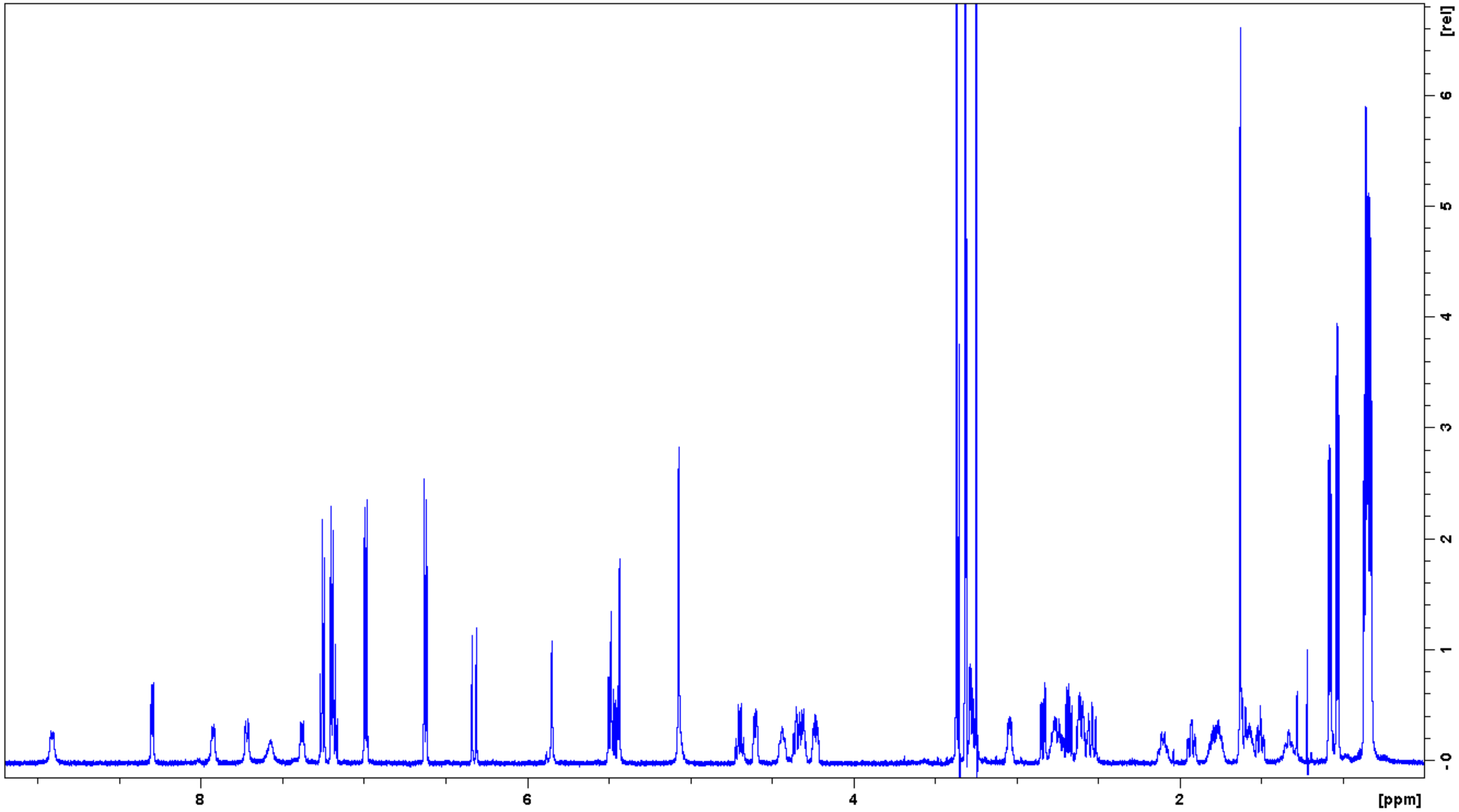

Figure S3. ^{1}H NMR Spectrum of [Leu[1]]MC-LY (**1**) in CD_3OH (600 MHz).

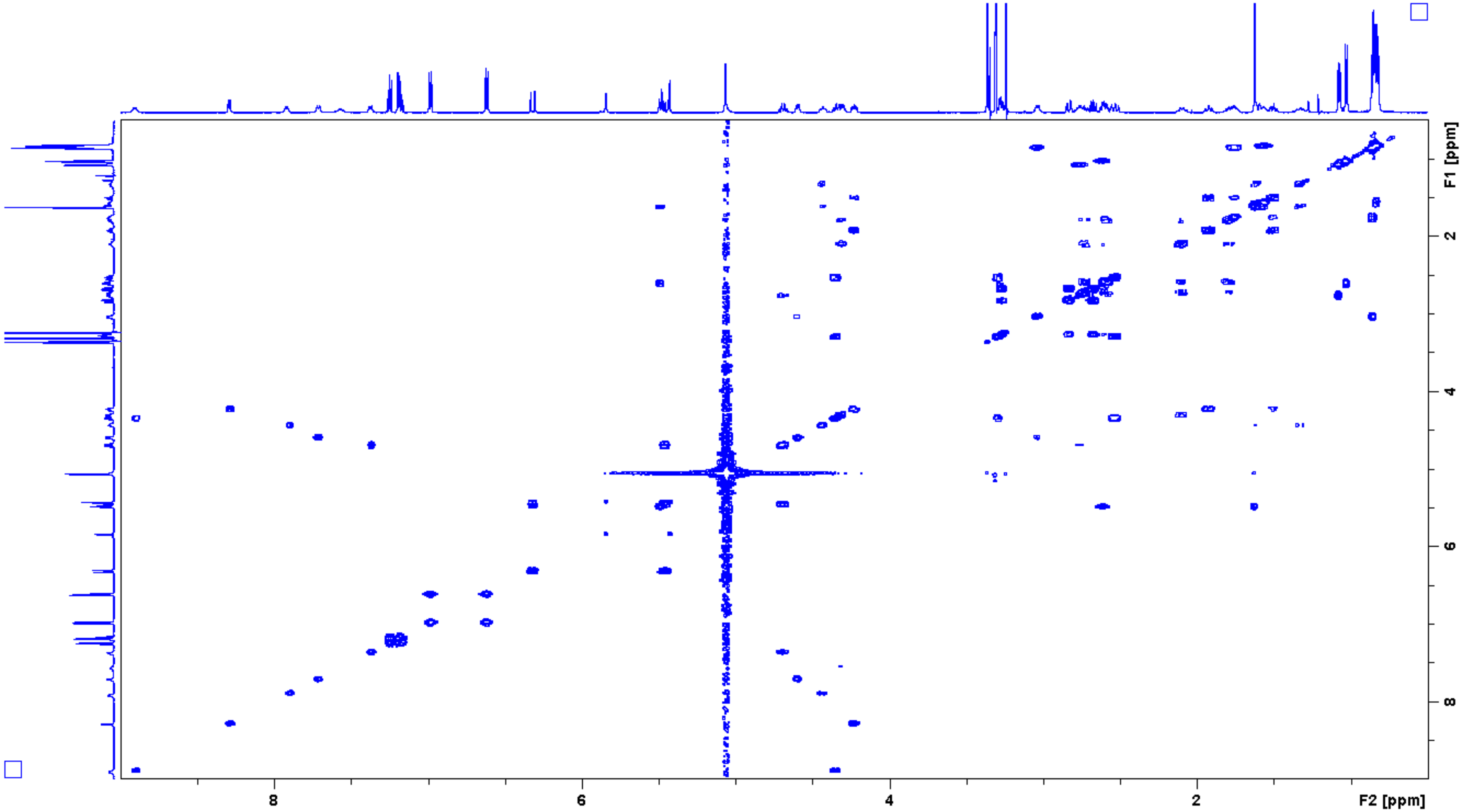

Figure S4. COSY NMR Spectrum of [Leu[1]]MC-LY (**1**) in CD_3OH (600 MHz).

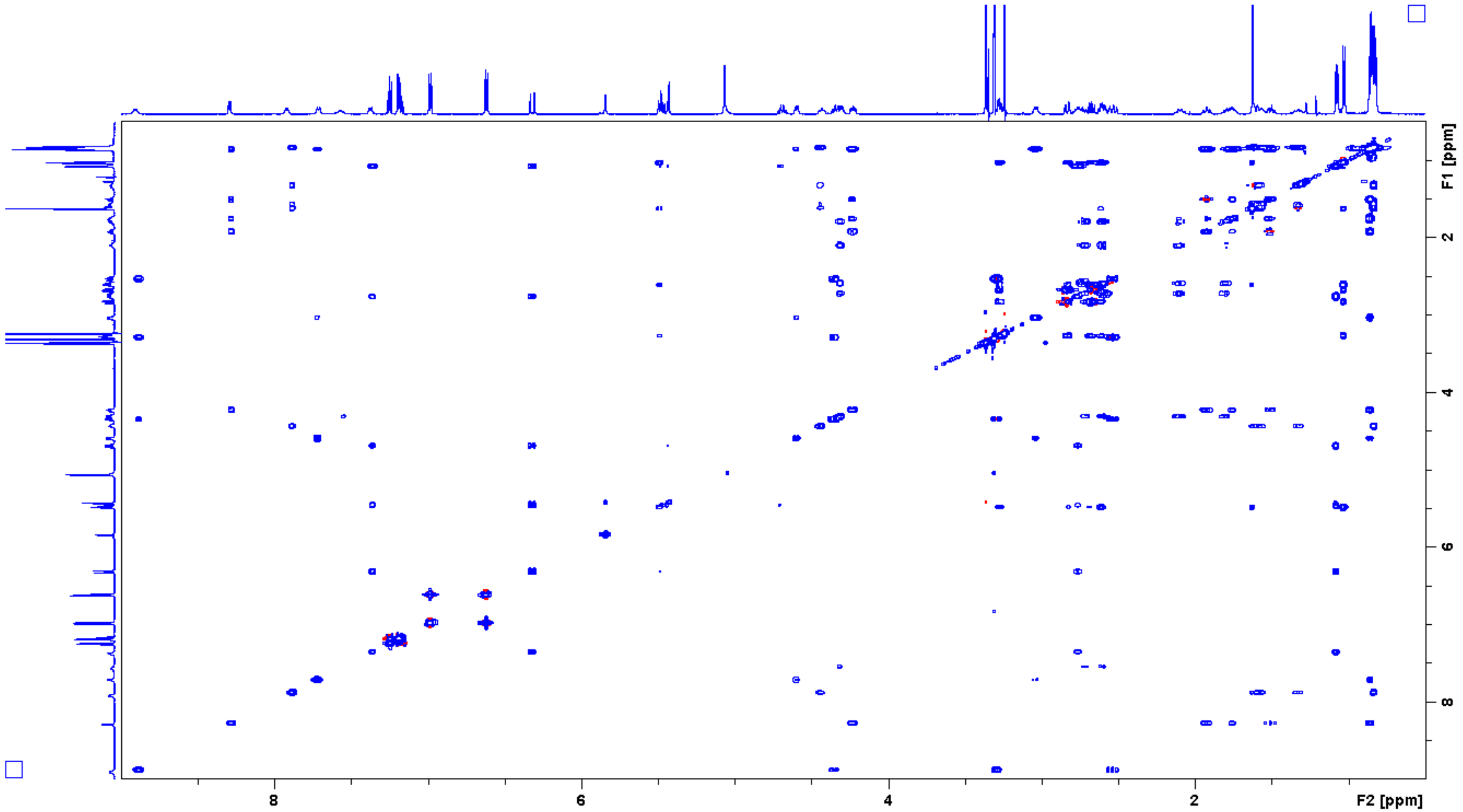

Figure S5. DIPSI NMR Spectrum of [Leu¹]MC-LY (**1**) in CD_3OH (600 MHz).

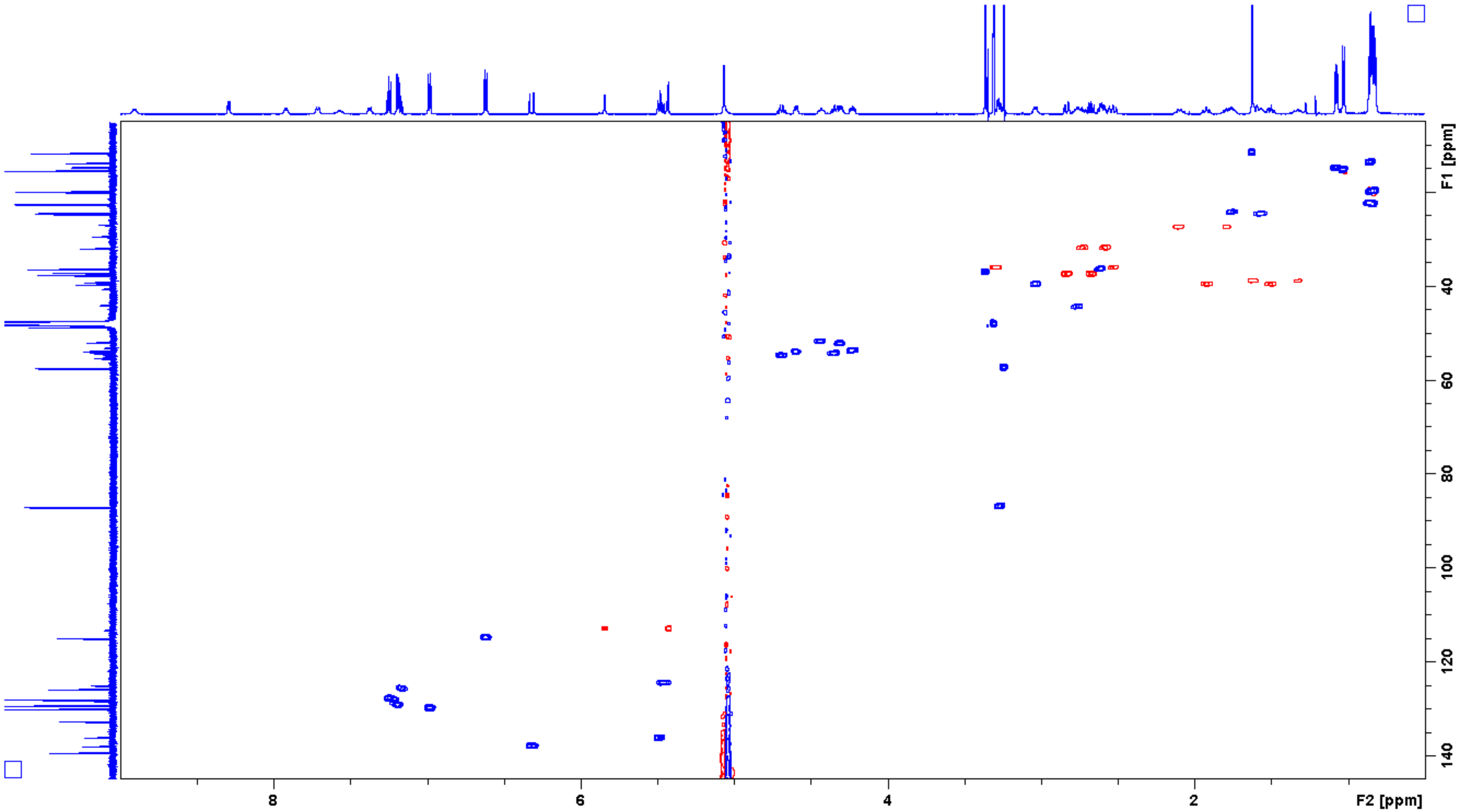

Figure S6. Edited HSQC NMR Spectrum of [Leu[1]]MC-LY (**1**) in CD_3OH (600 MHz).

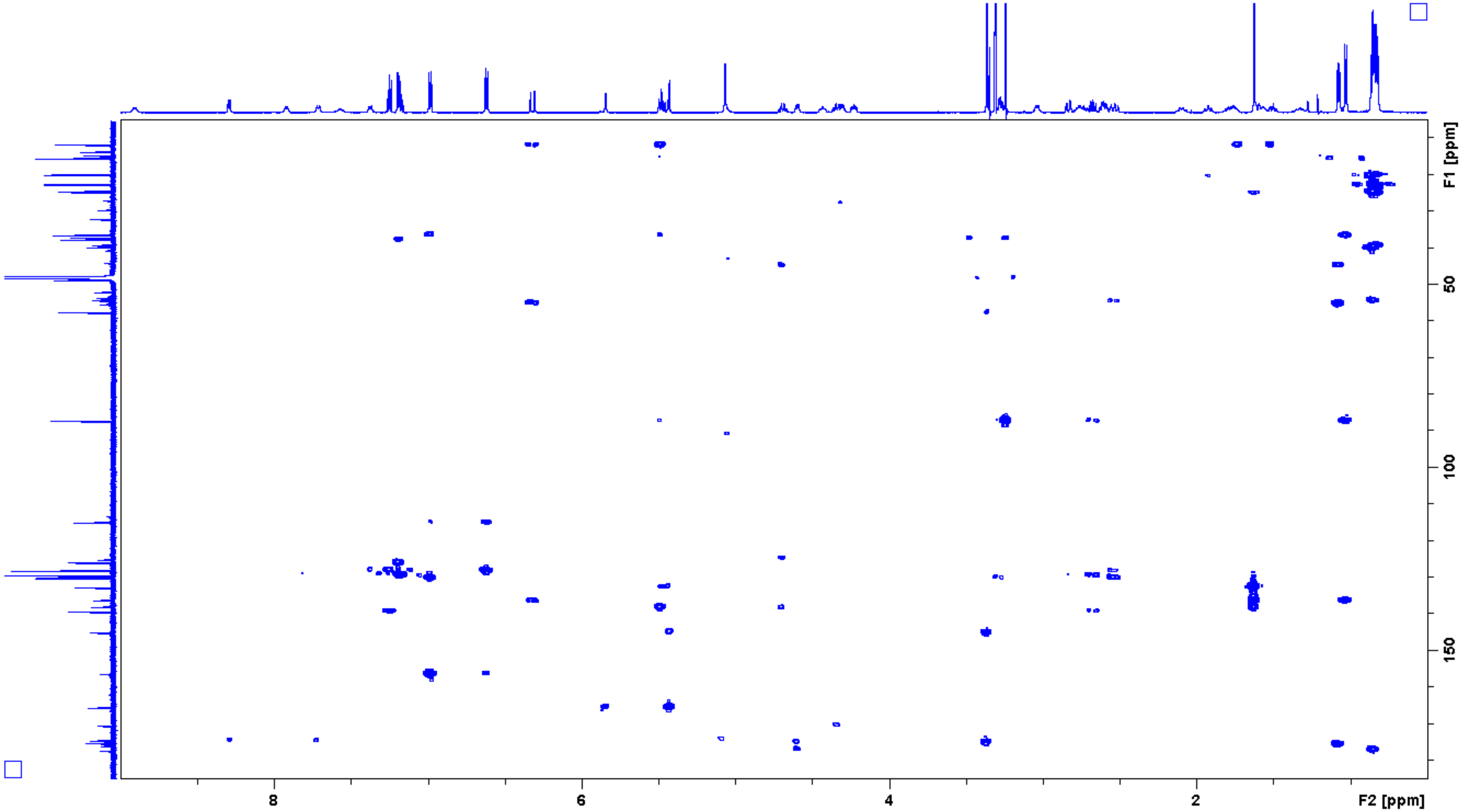

Figure S7. HMBC NMR Spectrum of [Leu[1]]MC-LY (**1**) in CD_3OH (600 MHz).

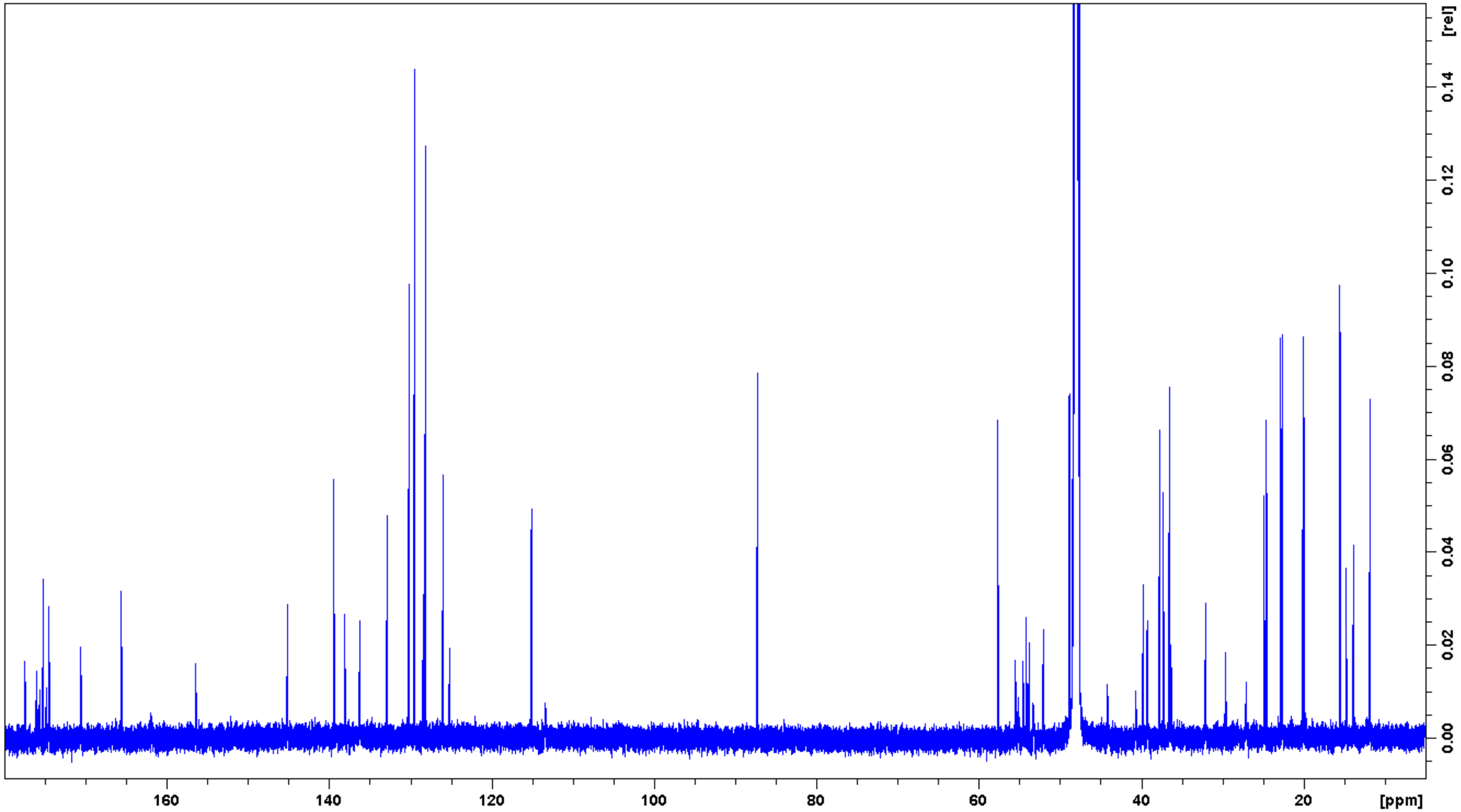

Figure S8. ^{13}C NMR Spectrum of [Leu[1]]MC-LY (**1**) in CD_3OH (176 MHz).

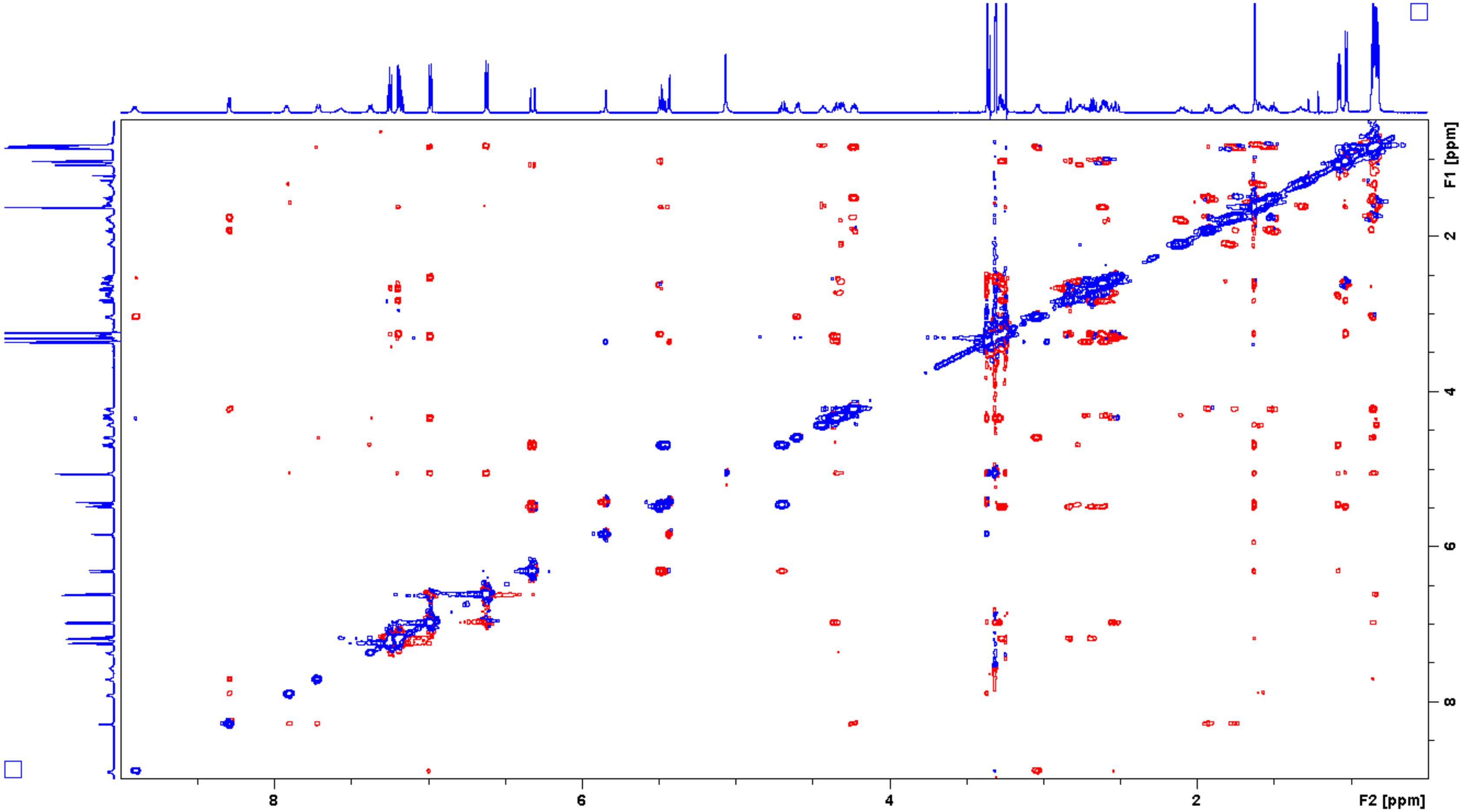

Figure S9. ROESY NMR Spectrum of [Leu[1]]MC-LY (**1**) in CD_3OH (600 MHz).

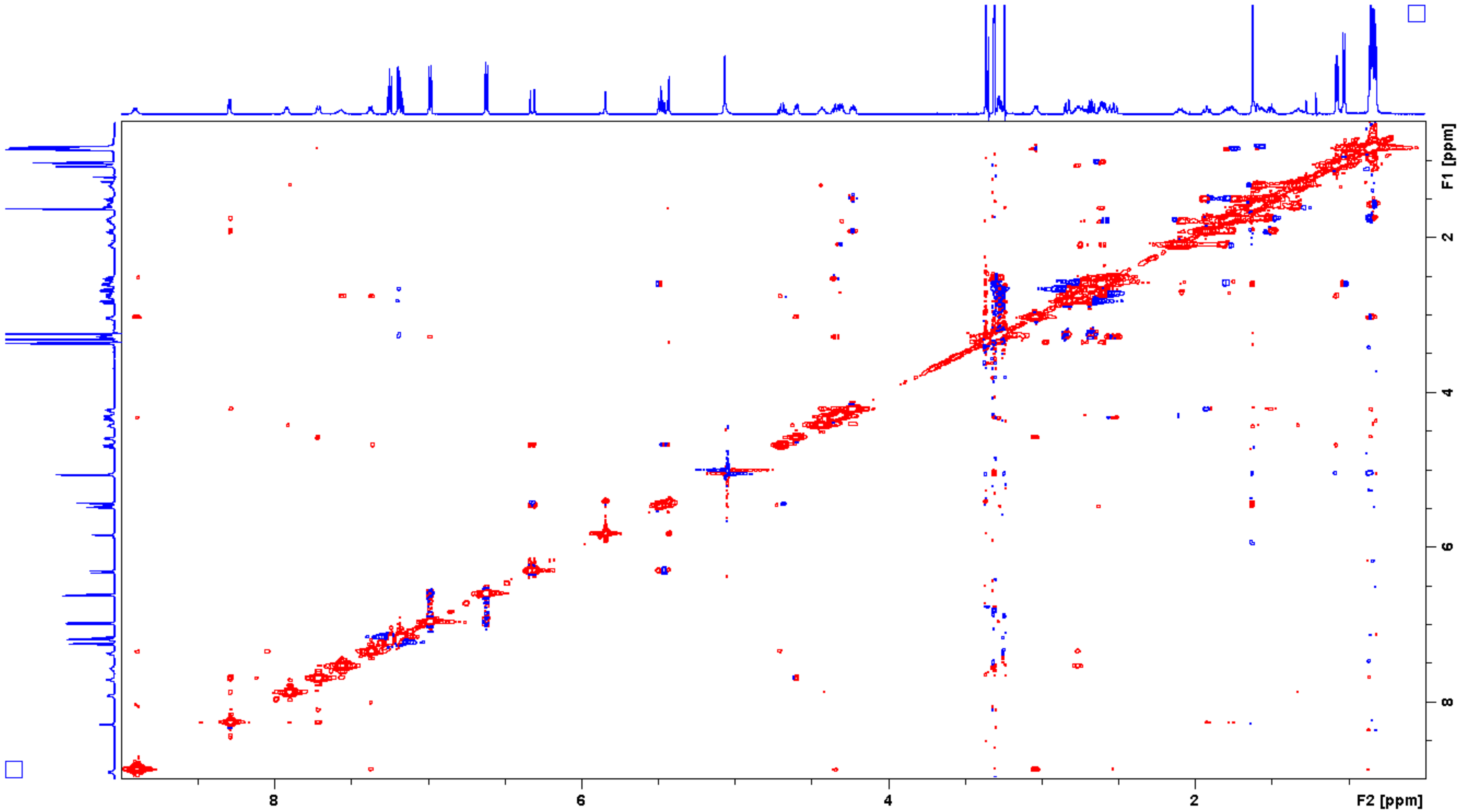

Figure S10. NOESY NMR Spectrum of [Leu[1]]MC-LY (**1**) in CD_3OH (600 MHz).

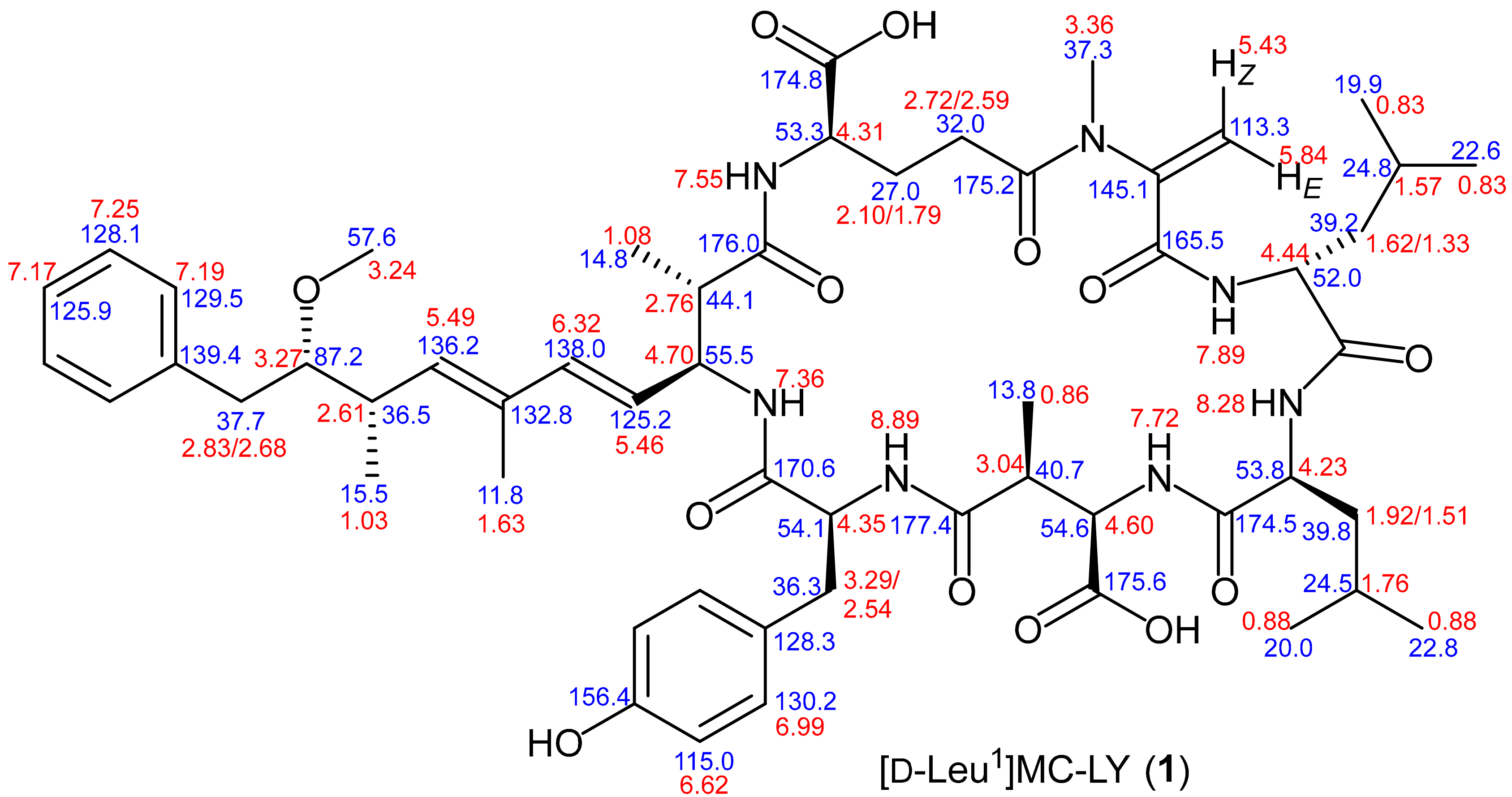

Figure S11. ^{1}H (red text) and ^{13}C (blue text) NMR chemical shift assignments for [D-Leu[1]]MC-LY (**1**) in CD_3OH, taken from Table 1, overlaid on the 2-dimensional chemical structure of **1**.

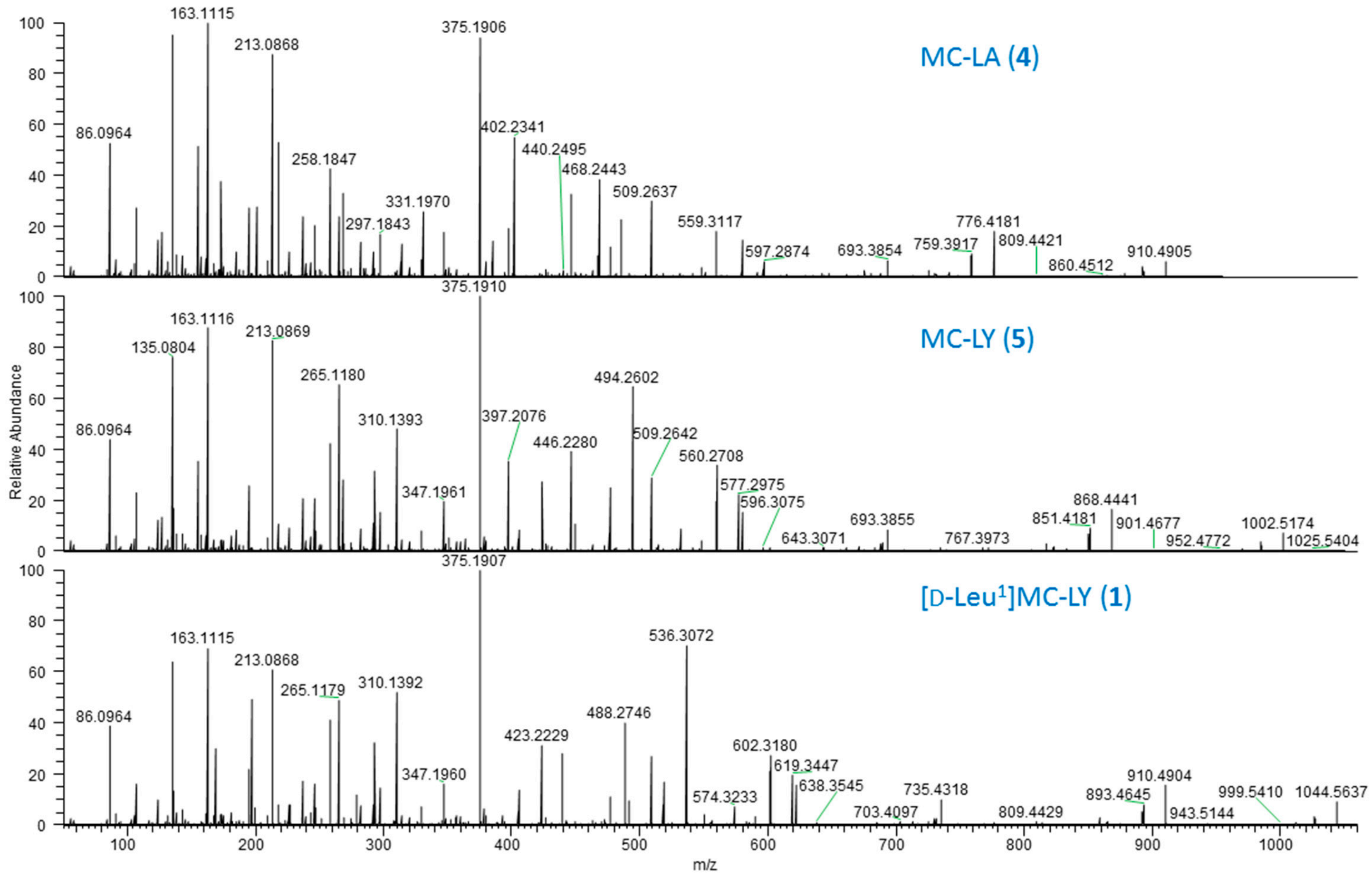

Figure S12. LC–HRMS/MS spectra of authentic MC-LA (**4**) and MC-LY (**5**), and of [Leu[1]]MC-LY (**1**) from *M. aeruginosa* CPCC-464 in positive ionization mode.

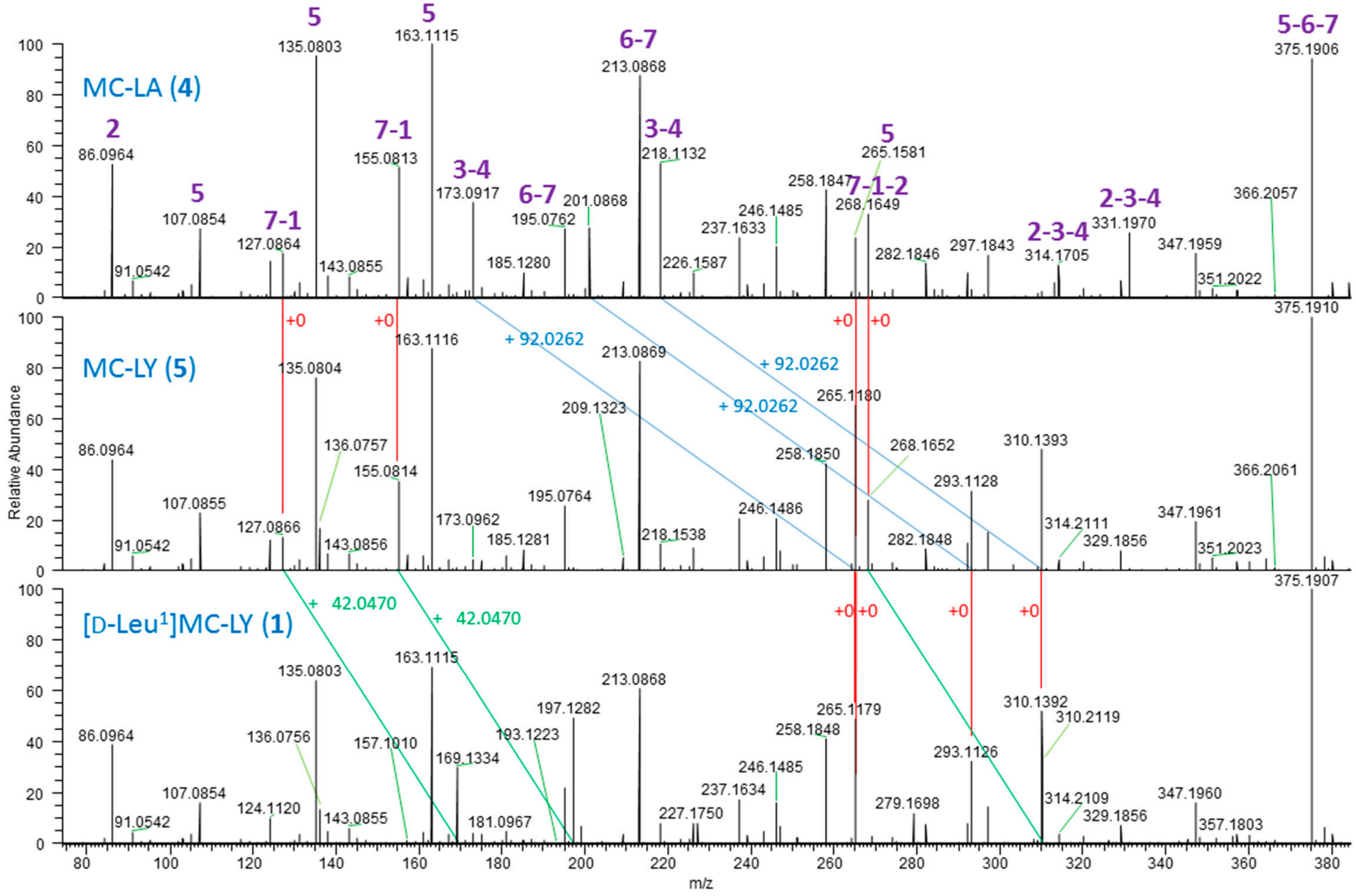

Figure S13. Expansion of the LC–HRMS/MS spectra of [M + H]$^+$ for MC-LA (**4**), MC-LY (**5**), and [Leu[1]]MC-LY (**1**) obtained in positive ionization mode (Figure S13). Product ions that change in *m*/*z* between any of the three spectra marked with red (no change), blue (+ 92.0262) or green (+ 42.0470). These changes in mass correspond to the exact mass difference between **4** and **5** (*m*/*z* 92.0262, replacing Ala[4] with Tyr[4]), and between **5** and **1** (*m*/*z* 42.0470, replacing D-Ala[1] with D-Leu[1]). The bold purple numbers indicate the microcystin amino acid residue numbers proposed to be responsible for selected product ions (see also Table 2).

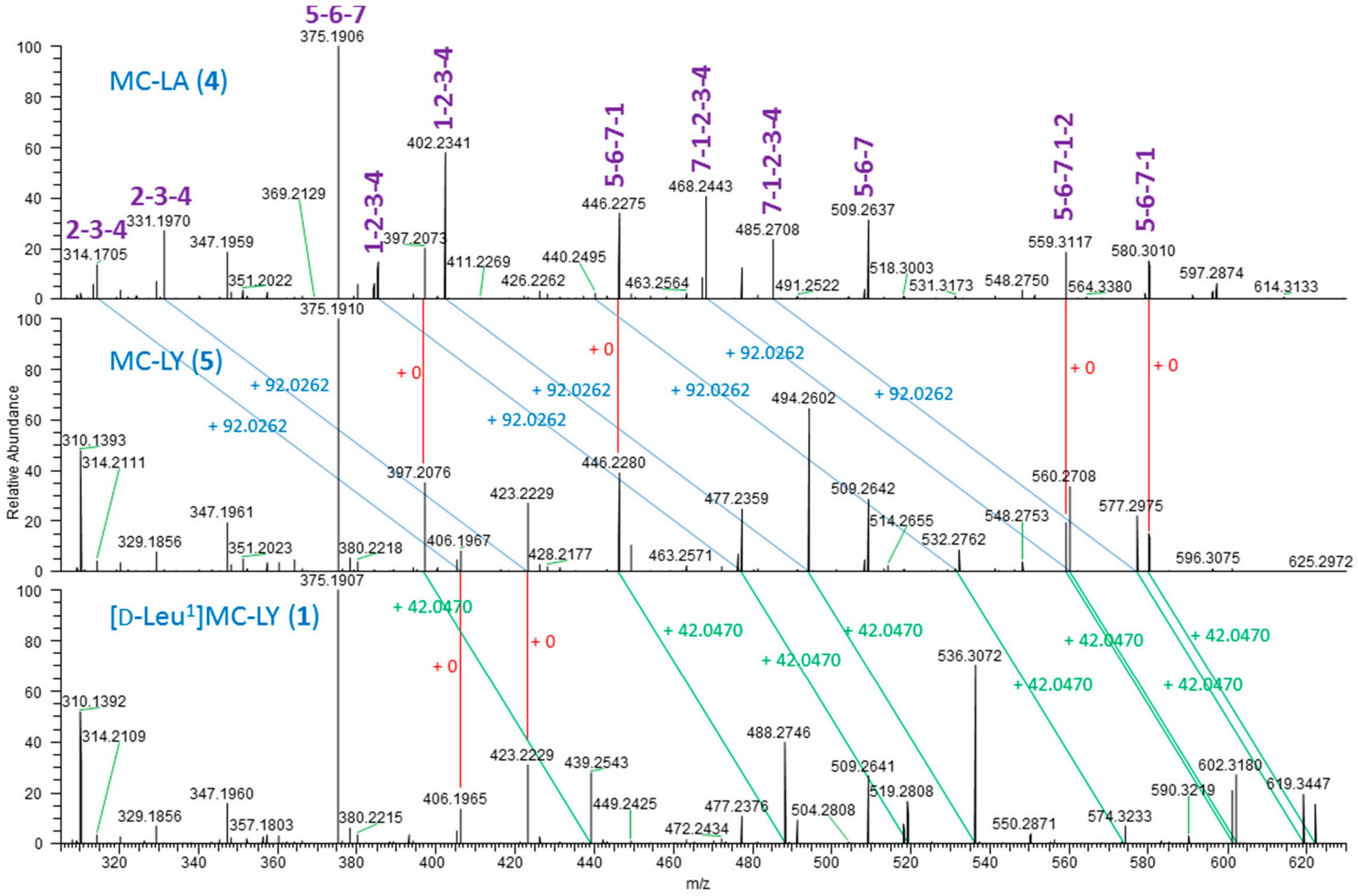

Figure S14. Expansion of the LC–HRMS/MS spectra of [M + H]⁺ for MC-LA (**4**), MC-LY (**5**), and [Leu¹]MC-LY (**1**) obtained in positive ionization mode (Figure S13). Product ions that change in *m*/*z* between any of the three spectra marked with red (no change), blue (+ 92.0262) or green (+ 42.0470). These changes in mass correspond to the exact mass difference between **4** and **5** (*m*/*z* 92.0262, replacing Ala⁴ with Tyr⁴), and between **5** and **1** (*m*/*z* 42.0470, replacing D-Ala¹ with D-Leu¹). The bold purple numbers indicate the microcystin amino acid residue numbers proposed to be responsible for selected product ions (see also Table 2).

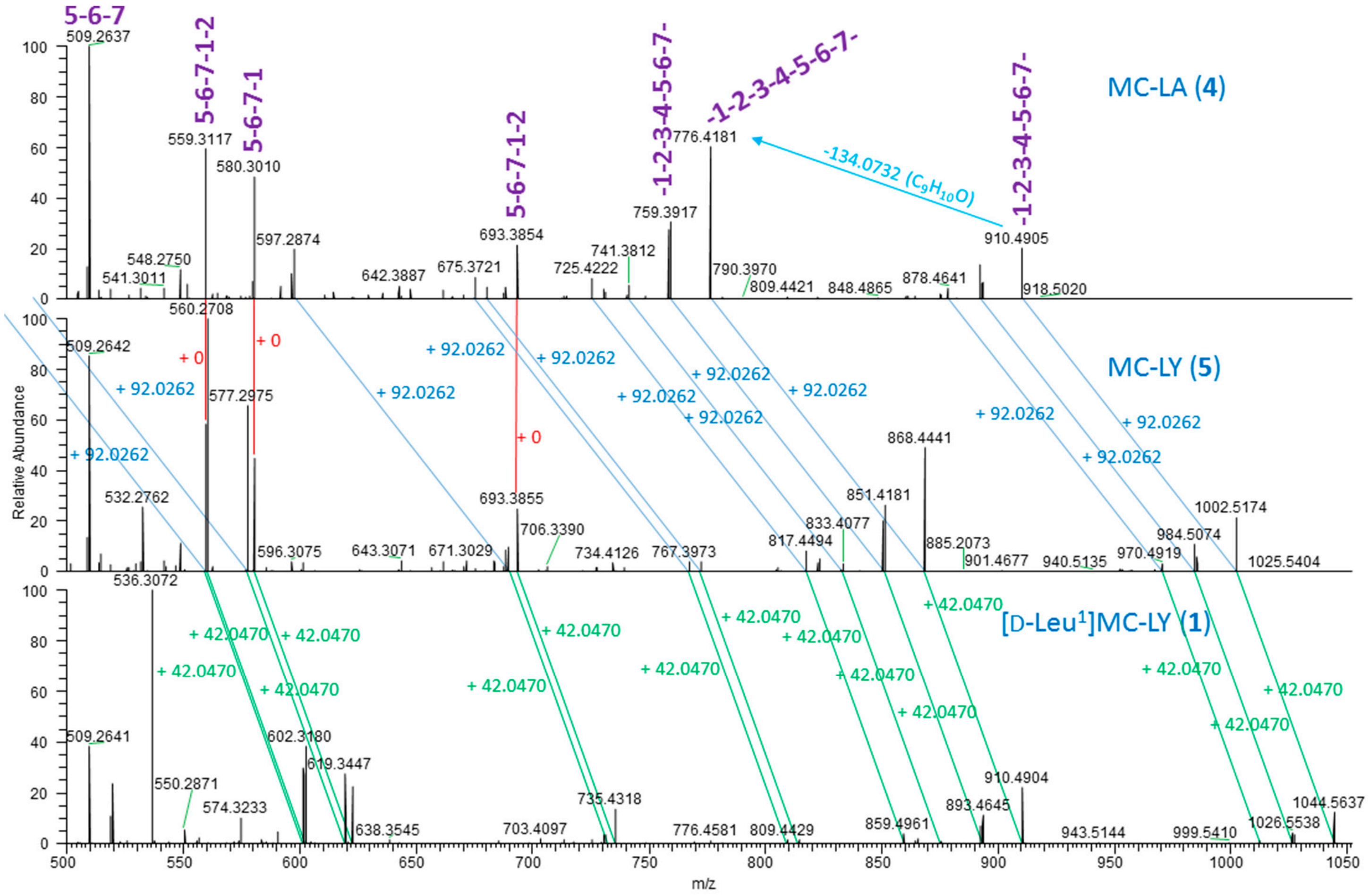

Figure S15. Expansion of the LC–HRMS/MS spectra of [M + H]$^+$ for MC-LA (**4**), MC-LY (**5**), and [Leu^1]MC-LY (**1**) obtained in positive ionization mode (Figure S13). Product ions that change in *m*/*z* between any of the three spectra marked with red (no change), blue (+ 92.0262) or green (+ 42.0470). These changes in mass correspond to the exact mass difference between **4** and **5** (*m*/*z* 92.0262, replacing Ala^4 with Tyr^4), and between **5** and **1** (*m*/*z* 42.0470, replacing D-Ala^1 with D-Leu^1). The bold purple numbers indicate the microcystin amino acid residue numbers proposed to be responsible for selected product ions (see also Table 2).

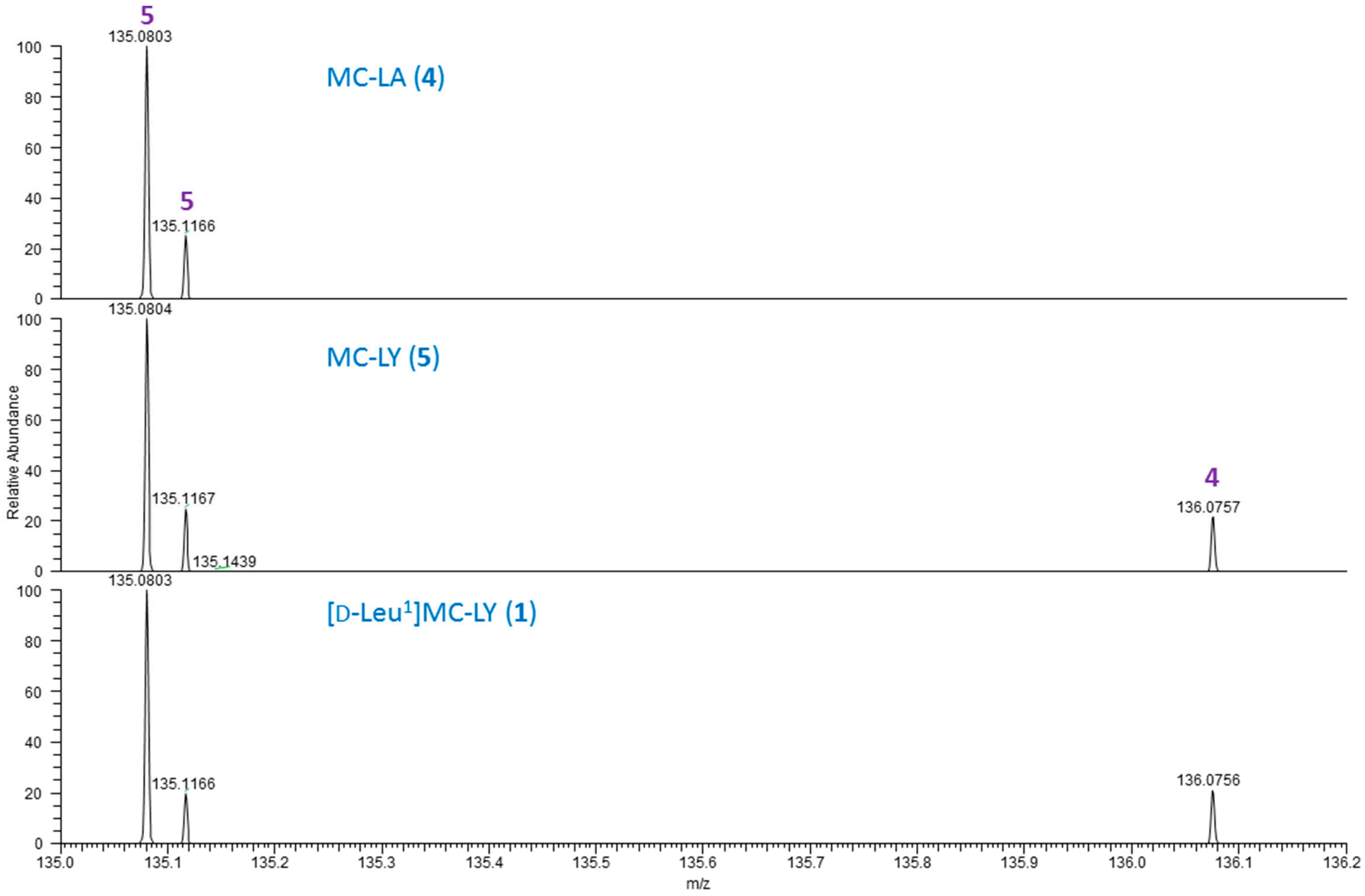

Figure S16. Expansion of the LC–HRMS/MS spectra of [M + H]$^+$ for MC-LA (**4**), MC-LY (**5**), and [Leu[1]]MC-LY (**1**) obtained in positive ionization mode (Figure S13), showing the region at *m*/*z* 135.0–136.2 containing the Adda and Tyr fragments (the latter product ion only being present in MC-LY (**5**) and [Leu[1]]MC-LY (**1**)). The bold purple numbers indicate the microcystin amino acid residue numbers proposed to be responsible for selected product ions (see also Table 2).

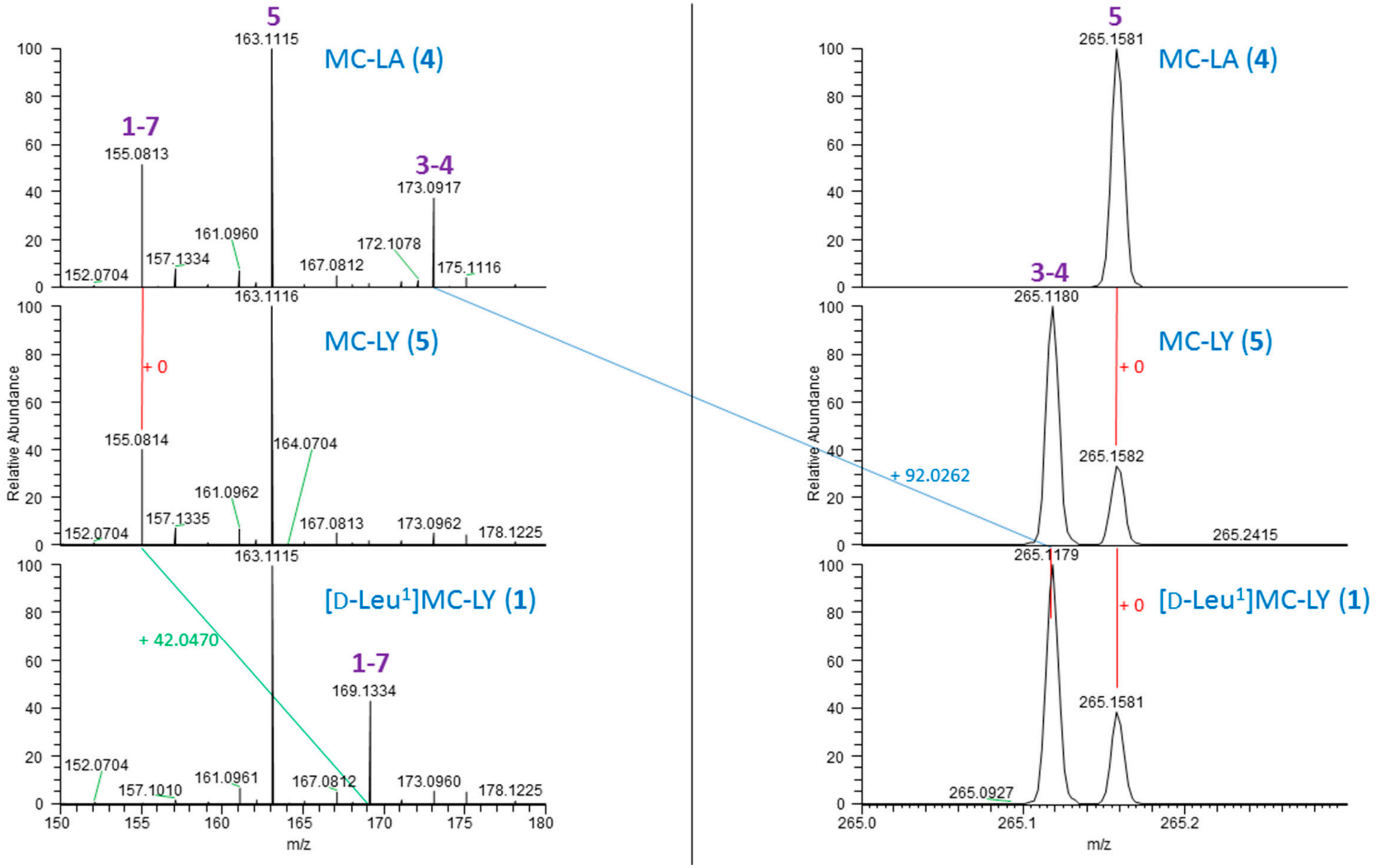

Figure S17. Expansion of the LC–HRMS/MS spectra of [M + H]+ for MC-LA (**4**), MC-LY (**5**), and [Leu¹]MC-LY (**1**) obtained in positive ionization mode (Figure S13), showing *m*/*z* 150–180 and 265.0–265.3 and revealing the presence of two distinct product ions at ca 265.1 in **1** and **5**. Product ions that change in *m*/*z* between any of the three spectra are marked with red (no change), blue (+ 92.0262) or green (+ 42.0470). These changes in mass correspond to the exact mass difference between **4** and **5** (*m*/*z* 92.0262, replacing Ala⁴ with Tyr⁴), and between **5** and **1** (*m*/*z* 42.0470, replacing D-Ala¹ with D-Leu¹). The bold purple numbers indicate the microcystin amino acid residue numbers proposed to be responsible for selected product ions (see also Table 2).

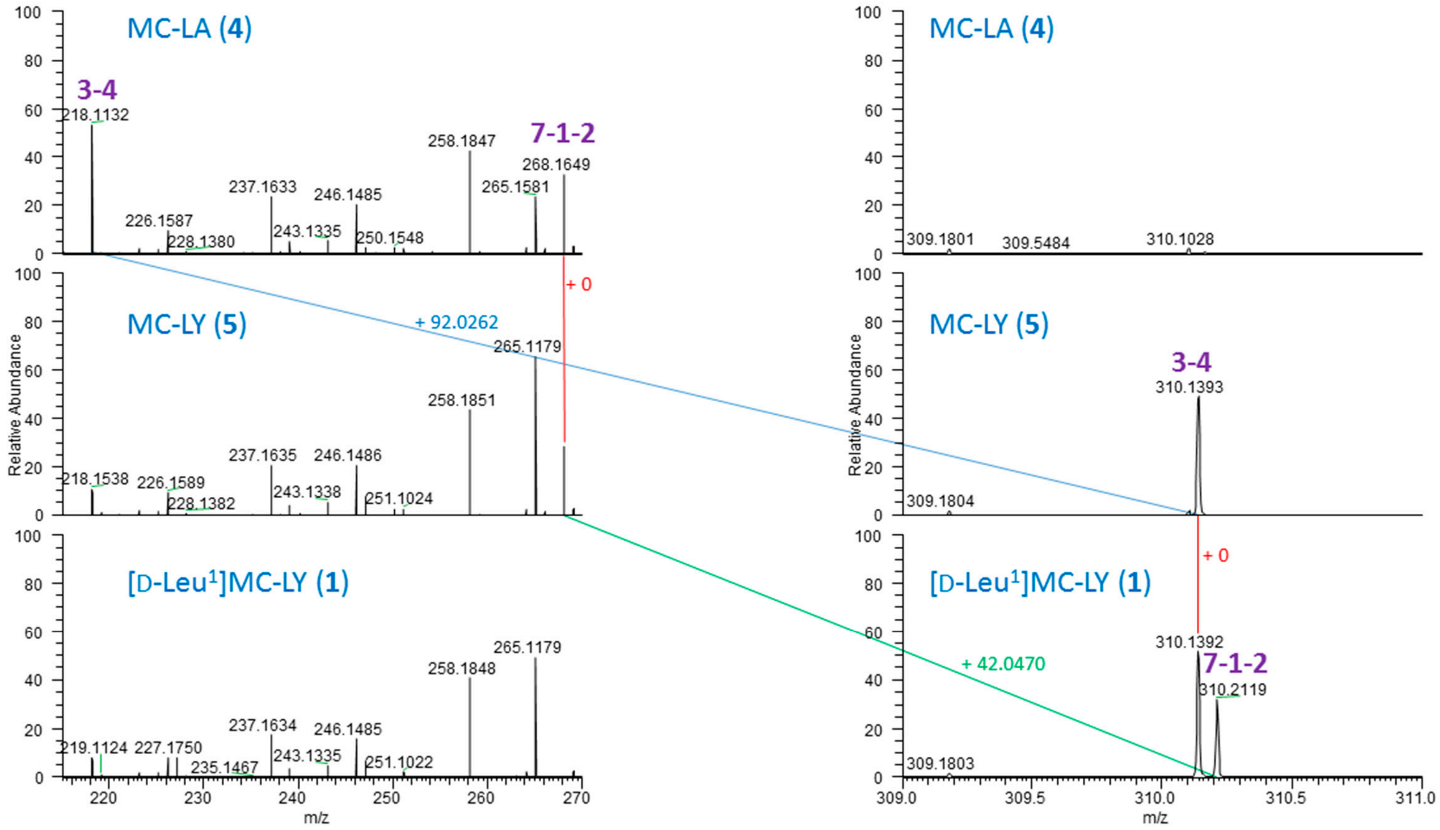

Figure S18. Expansion of the LC–HRMS/MS spectra of [M + H]$^+$ for MC-LA (**4**), MC-LY (**5**), and [Leu[1]]MC-LY (**1**) obtained in positive ionization mode (Figure S13), showing *m*/*z* 215–270 and 309–311 and revealing the presence of two distinct product ions at ca *m*/*z* 310 in **1**. Product ions that change in *m*/*z* between any of the three spectra are marked with red (no change), blue (+ 92.0262) or green (+ 42.0470). These changes in mass correspond to the exact mass difference between **4** and **5** (*m*/*z* 92.0262, replacing Ala[4] with Tyr[4]), and between **5** and **1** (*m*/*z* 42.0470, replacing D-Ala[1] with D-Leu[1]). The bold purple numbers indicate the microcystin amino acid residue numbers proposed to be responsible for selected product ions (see also Table 2).

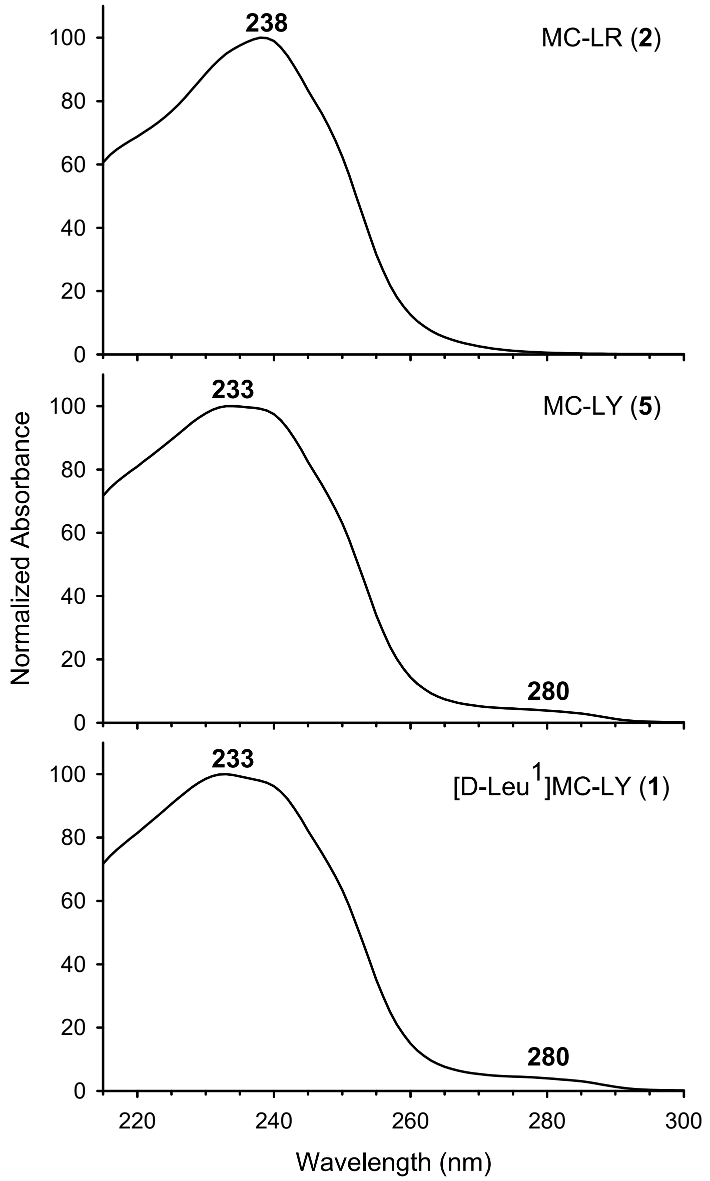

Figure S19. UV spectra obtained from isocratic LC–UV analysis of authentic MC-LR (**2**) and MC-LY (**5**), and purified [Leu[1]]MC-LY (**1**) isolated during this study.

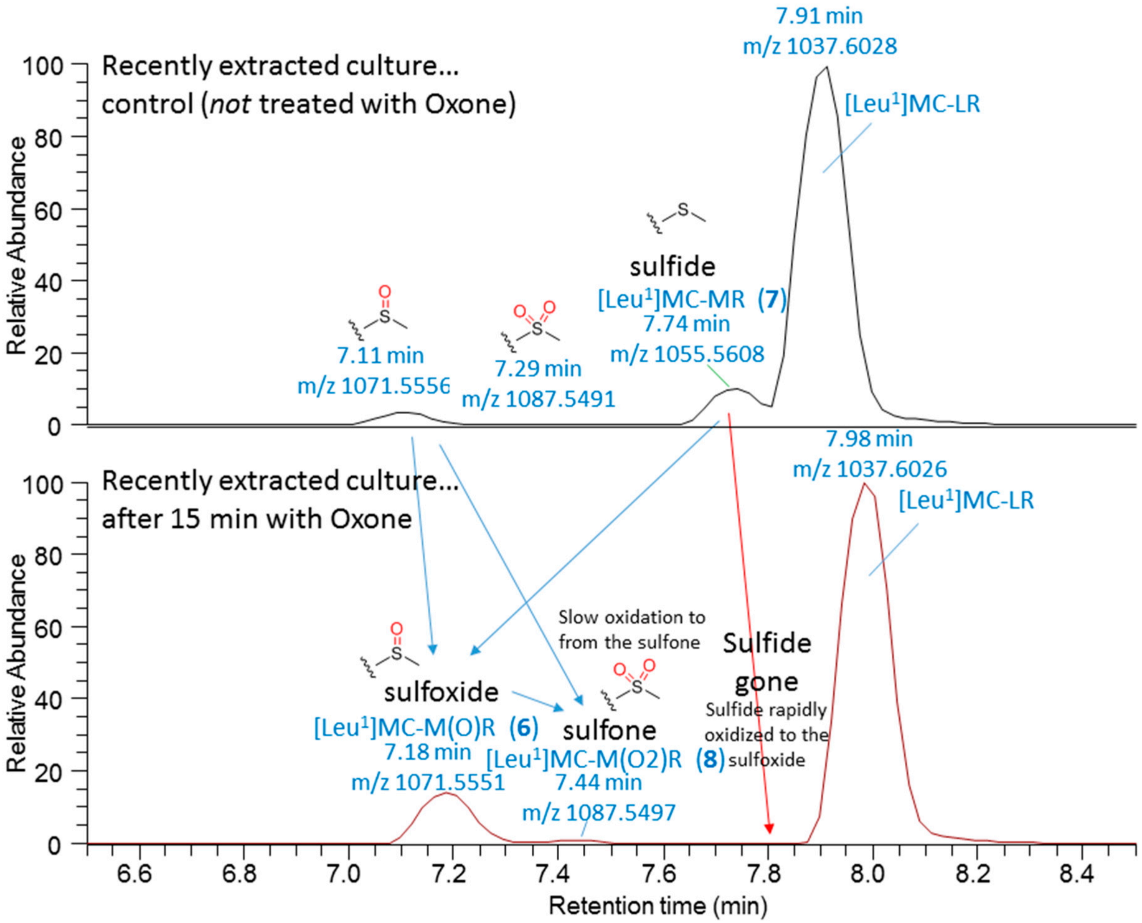

Figure S20. Positive ionization mode full scan LC-HRMS chromatograms of an extract of *M. aeruginosa* CPCC-464 extracted at *m/z* for [Leu[1]]MC-LR and **6**–**8**, before and shortly after treatment with the oxidant Oxone. Note the complete and rapid conversion of the methionine group in sulfide-**7** to form sulfoxide-**6**, and the much slower oxidation of sulfoxide-**6** to sulfone-**8**. Trace levels of sulfone-**8** were present prior to addition of the oxidant.

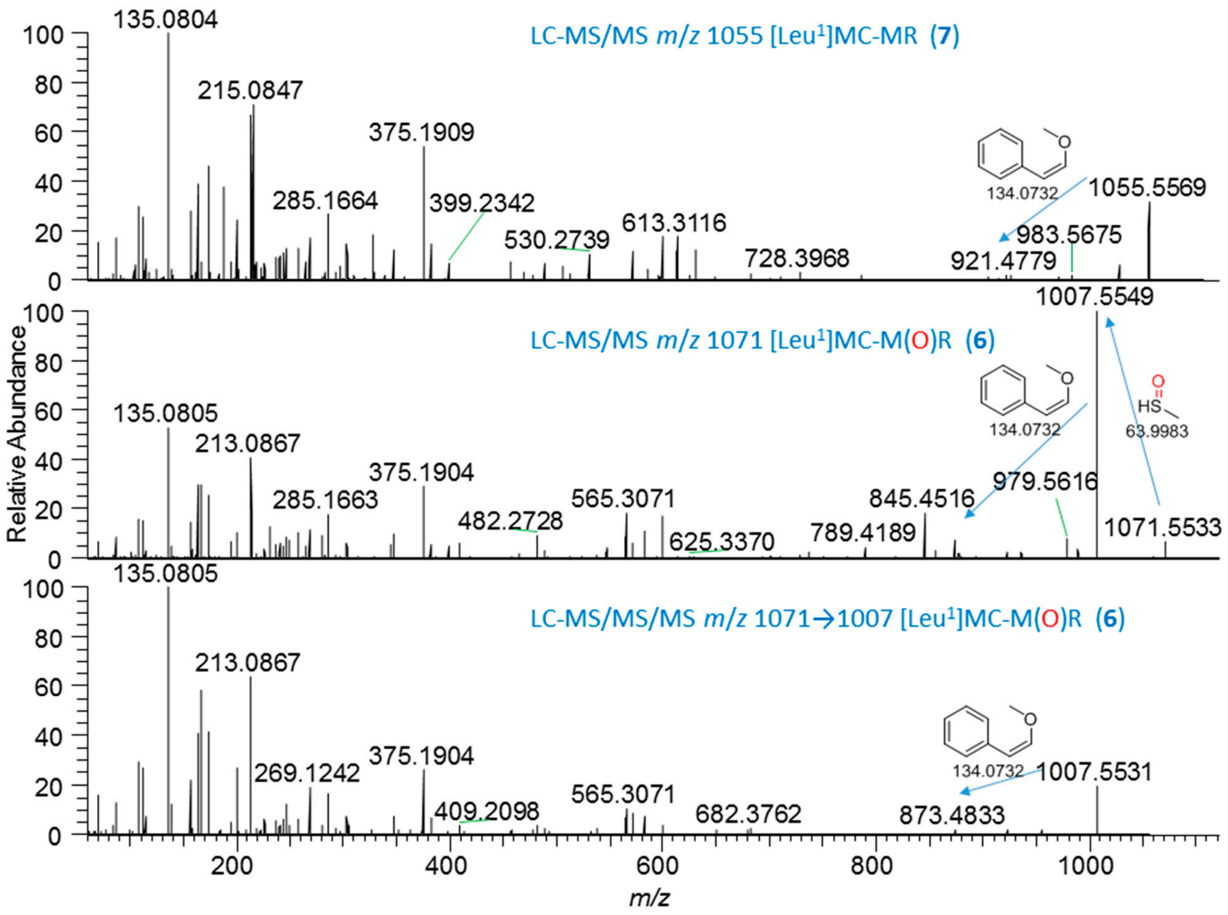

Figure S21. LC–HRMS/MS spectra of $[M + H]^+$ of [Leu[1]]MC-MR (**7**) (top) and its corresponding sulfoxide [Leu[1]]MC-M(O)R (**6**) (middle), obtained from an extract of *M. aeruginosa* CPCC-464. Note the prominent neutral loss of CH_4OS (*m/z* 63.9983) for **6** that is characteristic of methionine sulfoxides. The bottom spectrum is the LC–HRMS/MS spectrum of the $[M + H]^+$ product ion at *m/z* 1007.5 of sulfoxide-**7** using in-source fragmentation.

Table S1. Comparison[a] of the ^{13}C chemical shift assignments for **1** in CD_3OH with those reported for **3** in CD_3OD.[1]

Unit	Position	1, δ_C	3, δ_C	Diff.
D-Leu^{1}	1	ND	175.1	N/A
	2	52.0	52.9	0.9
	3	39.2	40.9	1.7
	4	24.8	26.1	1.3
	4-Me	19.9	21.2	1.3
	5	22.6	23.6	1.0
Leu^{2}	1	174.5	175.4	0.9
	2	53.8	54.9	1.1
	3	39.8	40.8	1.0
	4	24.5	25.9	1.4
	4-Me	20.0	21.3	1.3
	5	22.8	23.7	0.9
D-Masp^{3}	1.0	175.6	176.4	0.8
	2	54.6	56.9	2.3
	3	40.7	42.1	1.4
	3-Me	13.8	15.5	1.7
	4	177.4	179.3	1.9
Tyr^{4}/ Arg^{4}	1	170.6	172.1	1.5
	2	54.1	53.2	-0.9
Adda^{5}	1	176.0	176.8	0.8
	2	44.1	45.3	1.2
	2-Me	14.8	16.0	1.2
	3	55.5	56.8	1.3
	4	125.2	126.7	1.5
	5	138.0	139.0	1.0
	6	132.8	133.9	1.1
	6-Me	11.8	12.9	1.1
	7	136.2	137.1	0.9
	8	36.5	37.7	1.2
	8-Me	15.5	16.5	1.0
	9	87.2	88.5	1.3
	9-OMe	57.6	58.8	1.2
	10	37.7	39.0	1.3
	11	139.4	140.6	1.2
	12/16	129.5	130.5	1.0
	13/15	128.1	129.2	1.1
	14	125.9	127.1	1.2
D-Glu^{6}	1	174.8	177.2	2.4
	2	53.3	55.4	2.1
	3	27.0	28.3	1.3
	4	32.0	33.2	1.2

	5	175.2	176.7	1.5
Mdha[7]	1	165.5	166.4	0.9
	2	145.1	146.3	1.2
	2-NMe	37.3	38.4	1.1
	3	113.3	114.7	1.4
			**Mean**	**1.2**
			**SD**	**0.5**

[a] Note, the reference value for chemical shift calibration was not reported for **3**.[1] Values differing from the mean chemical shift difference (1.2 ppm) by more than the standard deviation (0.5 ppm) are shown in red (higher) or blue (lower) text. Only chemical shifts for C-1 and C-2 are shown for amino acid-4, because this is the only residue that differs between **1** and **3**, the former containing tyrosine and the latter containing arginine at position-4. Only minor differences were observed, for: carbon atoms close to the carboxyl groups in the D-Masp[3] and D-Glu[6] residues, attributable to a different degree of carboxyl protonation between the two samples; and at C-2 of position-4, attributable to the different amino acid side-chains present at this position.

References

1. Schripsema, J.; Dagnino, D. Complete assignment of the NMR spectra of [D-Leu[1]]-microcystin-LR and analysis of its solution structure. *Magn. Reson. Chem.* **2002**, *40*, 614–617.
